# Margin status, re-excision, and completion mastectomy after oncoplastic versus conventional breast-conserving surgery for breast cancer: a systematic review and meta-analysis

**DOI:** 10.3389/fonc.2026.1934279

**Published:** 2026-09-15

**Authors:** Na Hou, Lu Ye, Wei Huang, Yanmei Yin, Junwen Peng

**Affiliations:** Department of General Surgery, The First People’s Hospital of Jiande, Jiande, China

**Keywords:** breast cancer, completion mastectomy, oncoplastic breast-conserving surgery, re-excision, surgical margin

## Abstract

**Background:**

Oncoplastic breast-conserving surgery (OBCS) may permit wider excision within a breast-conserving approach, but its effects on margin control and subsequent surgery remain uncertain.

**Methods:**

We searched PubMed/MEDLINE, Embase, Web of Science Core Collection, Scopus, and the Cochrane Library from inception through 25 June 2026 for comparative studies of OBCS versus conventional breast-conserving surgery (BCS). Primary outcomes were initial positive margin, re-excision, completion/conversion mastectomy, and any margin-related reoperation. Secondary outcomes were negative/clear margin, recurrence, and complications. Risk ratios (RRs) with 95% confidence intervals (CIs) were pooled using random-effects Mantel–Haenszel models. Methodological quality was assessed using the Newcastle–Ottawa Scale. The protocol was registered in PROSPERO (CRD420261439265).

**Results:**

Forty-eight comparative studies comprising 124,903 BCS observations were included (28,085 OBCS; 96,818 conventional BCS). Compared with conventional BCS, OBCS was associated with lower risks of initial positive margin (RR 0.57, 95% CI 0.40–0.80), re-excision (RR 0.59, 95% CI 0.46–0.77), completion/conversion mastectomy (RR 0.62, 95% CI 0.43–0.90), and any margin-related reoperation (RR 0.63, 95% CI 0.51–0.78). OBCS was also associated with a higher likelihood of achieving negative/clear margins (RR 1.07, 95% CI 1.04–1.11). No significant between-group differences were observed in local recurrence/ipsilateral breast tumor recurrence or locoregional recurrence. Overall complication risk was higher in the primary analysis, but the association was attenuated to borderline statistical significance after exclusion of registry or administrative-database cohorts.

**Conclusions:**

OBCS was associated with more favorable margin-related outcomes and fewer margin-driven reoperations, without a detectable increase in recurrence. These findings support selective use of OBCS as a margin-control and breast-preservation strategy in appropriately selected patients. However, complication estimates should be interpreted cautiously because reporting and ascertainment varied across study designs.

**Systematic review registration:**

https://www.crd.york.ac.uk/PROSPERO/view/CRD420261439265, identifier CRD420261439265.

## Introduction

The success of breast-conserving treatment depends not only on avoiding mastectomy at the initial operation but also on achieving adequate margins, preserving breast form, and avoiding further surgery. This issue has clear population-level relevance because breast cancer remains the most frequently diagnosed cancer in women worldwide, and these surgical decisions affect a large and heterogeneous patient population requiring individualized treatment planning (1). Contemporary data have also challenged the assumption that more extensive breast removal necessarily reduces breast cancer mortality. In a large population-based cohort study, bilateral mastectomy markedly reduced contralateral breast cancer but did not reduce breast cancer mortality compared with lumpectomy or unilateral mastectomy (2). Together with individual patient-level evidence that radiotherapy after breast-conserving surgery reduces recurrence and breast cancer mortality, these findings support a contemporary surgical priority: breast conservation should be both oncologically sound and durable (3).

Margin status is central to this pathway. The Society of Surgical Oncology–American Society for Radiation Oncology consensus guideline defined “no ink on tumor” as an adequate negative margin for invasive breast cancer treated with whole-breast irradiation, whereas the Society of Surgical Oncology–American Society for Radiation Oncology–American Society of Clinical Oncology guideline recommended a 2-mm margin for ductal carcinoma *in situ* treated with whole-breast irradiation (4, 5). These thresholds reduced unnecessary pursuit of wider margins but did not eliminate margin-driven reoperation. A meta-analysis found that reoperation rates declined after publication of the invasive cancer margin guideline, although the magnitude of this effect varied substantially across practice settings (6). More recent data on healthcare costs show that re-excision and conversion to mastectomy remain clinically and economically consequential after attempted breast-conserving surgery (7). Margin status is therefore not merely a pathological descriptor; it can determine the subsequent surgical course.

The initial operation provides the best opportunity to avoid this sequence. The randomized cavity-shave trial demonstrated that routine shave margins reduced positive margins and re-excision, showing that margin outcomes can be modified by operative strategy rather than being determined solely by tumor biology (8). OBCS extends this principle to a more complex surgical problem. By combining oncologic excision with immediate reshaping, volume displacement, volume replacement, or therapeutic mammoplasty, OBCS may permit wider excision than conventional BCS while mitigating the deformity that can follow large-volume lumpectomy. OBCS may be particularly useful when conventional breast conservation is technically or cosmetically challenging, including cases with a high tumor-to-breast volume ratio, extensive ductal carcinoma *in situ*, multifocal disease, tumors in cosmetically sensitive locations, or situations in which mastectomy is considered partly because standard lumpectomy would be inadequate or disfiguring. Pre-existing breast asymmetry may also favor OBCS when the tumor is located in the larger breast, because oncologic resection can be combined with reshaping or volume reduction to improve symmetry (9).

Despite increasing use, the evidence base for OBCS remains limited. A Cochrane Review concluded that although OBCS is widely used, the certainty of evidence remains limited because studies vary in comparator selection, case complexity, outcome reporting, and follow-up (10). A GRADE-based expert recommendation supported OBCS for suitable candidates but explicitly acknowledged very low certainty for many important outcomes and a lack of standardized patient-centered and clinical endpoints (11). Recent prospective and longitudinal studies have strengthened the evidence by showing that OBCS can enable breast conservation and provide patient-reported outcome data, but OBCS is still often evaluated as a broad reconstructive strategy rather than a specific margin-control intervention (12, 13).

This distinction is clinically important. When positive margins, close margins, re-excision, completion/conversion mastectomy, recurrence, complications, cosmetic outcomes, and satisfaction are combined under a broad label of “surgical outcomes,” clinically distinct steps after lumpectomy are obscured. A positive margin is not equivalent to a close margin, and re-excision is not equivalent to completion/conversion mastectomy. Similarly, a composite reoperation endpoint cannot identify which event drives the result. Even strong observational evidence, such as the nationwide Danish study reporting lower rates of re-excision and conversion to mastectomy after OBCS, does not establish whether the apparent benefit is consistent across each step of the margin-to-reoperation pathway (14).

We therefore conducted a margin-focused systematic review and meta-analysis comparing OBCS with conventional BCS in patients with breast cancer. The primary objective was to determine whether OBCS was associated with better initial margin control and fewer margin-driven reoperations. Recurrence, complications, and patient-reported or cosmetic outcomes were assessed to place these margin-related findings in the context of oncologic safety and surgical morbidity.

## Methods

### Protocol and reporting standard

This systematic review and meta-analysis was prospectively registered in the International Prospective Register of Systematic Reviews (PROSPERO; registration No. CRD420261439265). The review followed the Preferred Reporting Items for Systematic Reviews and Meta-Analyses (PRISMA) 2020 statement (15) and methodological guidance from the Cochrane Handbook for Systematic Reviews of Interventions (16). Eligibility criteria, outcomes, data extraction procedures, and analytical methods were prespecified before quantitative synthesis.

### Search strategy

Two reviewers independently searched PubMed/MEDLINE, Embase, Web of Science Core Collection, Scopus, and the Cochrane Library from database inception through 25 June 2026. No publication-year restrictions were applied, and no language restrictions were imposed at the database-search stage. The search combined controlled vocabulary and free-text terms for breast cancer, oncoplastic breast-conserving surgery, conventional BCS, surgical margins, re-excision, reoperation, and completion or conversion mastectomy. Search terms were adapted to each database. Reference lists of eligible articles and relevant reviews were also screened manually. Full electronic search strategies are provided in the **Supplementary Material**.

### Eligibility criteria

Comparative clinical studies were eligible if they included patients with breast cancer undergoing BCS, compared OBCS with conventional BCS, and reported at least one extractable margin- or reoperation-related outcome. Eligible designs included randomized trials, prospective or retrospective comparative cohort studies, case-control studies, propensity score-matched studies, and observational studies with adjusted analyses. Conventional BCS included standard lumpectomy, wide local excision, partial mastectomy, segmentectomy, or quadrantectomy without immediate oncoplastic reconstruction.

Studies were excluded if they were non-comparative, lacked a conventional BCS comparator, evaluated mastectomy or post-mastectomy reconstruction without a separate conventional BCS group, compared only different oncoplastic techniques, or provided no extractable margin or reoperation data. Case reports, reviews, editorials, letters, protocols, previous meta-analyses, and conference abstracts without sufficient data were also excluded. Only full-text articles published in English were eligible because full-text assessment was required to extract surgical techniques, margin definitions, reasons for reoperation, and outcome definitions. For duplicate or overlapping cohorts, we retained the most complete, most recent, or most informative dataset for the outcome of interest.

### Study selection

After duplicate records were removed, two reviewers independently screened titles and abstracts and then assessed the full texts of potentially eligible reports. Disagreements were resolved through discussion, with adjudication by a third reviewer when necessary. Reasons for exclusion after full-text review were recorded and summarized in the PRISMA flow diagram.

### Data extraction and outcomes

Two reviewers independently extracted data using a standardized form. Extracted information included study design, country, sample size, patient and tumor characteristics, surgical technique, comparator procedure, follow-up duration, and all prespecified outcomes. When both crude and matched or adjusted data were available, matched or adjusted data were prioritized when directly applicable to the outcome of interest.

The primary outcomes were initial positive surgical margin, re-excision, completion/conversion mastectomy, and any margin-related reoperation. Secondary outcomes included close margin, negative/clear margin, local recurrence or ipsilateral breast tumor recurrence, locoregional recurrence, overall complications, and cosmetic or patient-reported outcomes when extractable. Positive and close margins were extracted separately whenever definitions permitted. Close margin was defined according to the threshold used in each original study, most commonly <1 mm or <2 mm. Broader or combined definitions, such as close/involved margins, were handled in sensitivity or supplementary analyses rather than pooled with the primary close-margin analysis. For postoperative complications, we extracted complication type and severity classification as reported in the original studies, including Clavien–Dindo grading when reported. We also recorded whether reported complications were restricted to the index breast or included a contralateral symmetrizing procedure when this information was available.

### Quality assessment

Two reviewers independently assessed methodological quality. Non-randomized comparative studies were evaluated using the Newcastle–Ottawa Scale (NOS), which assesses selection, comparability, and outcome ascertainment. Higher NOS scores were interpreted as indicating lower risk of bias within the limitations of observational evidence. The randomized trial was assessed separately using the revised Cochrane risk-of-bias tool for randomized trials (RoB 2) because the NOS is not intended for randomized evidence. Disagreements were resolved by consensus or adjudication by a third reviewer.

### Statistical analysis

Meta-analyses were performed using Review Manager version 5.4.1. Dichotomous outcomes were pooled as risk ratios (RRs) with 95% confidence intervals (CIs). Because clinical and methodological heterogeneity was expected across studies with respect to patient selection, OBCS technique, comparator procedure, margin definition, reoperation policy, follow-up duration, and outcome ascertainment, random-effects Mantel–Haenszel models were used for all primary and sensitivity analyses.

Statistical heterogeneity was assessed using Cochran’s Q test and the I² statistic. A P value <0.10 for Cochran’s Q test or an I² value >50% was considered to indicate substantial heterogeneity. For each endpoint, event counts and denominators were extracted separately for the OBCS and conventional BCS groups. Because the original reports did not consistently use the same analytical unit, denominators were retained according to the unit reported in each study, including patient-, breast-, procedure-, or tumor-level denominators, as applicable.

Sensitivity analyses examined the influence of overlapping cohorts, alternative datasets, non-standard outcome definitions, data-coding caveats, and large registry-based or administrative-database studies. These analyses were treated as robustness checks rather than independent confirmatory analyses. Funnel plots were generated alongside the corresponding forest plots to visually assess small-study effects. Because several outcomes included fewer than 10 comparisons and multiple endpoints showed moderate-to-substantial heterogeneity, funnel plots were interpreted cautiously and formal tests for small-study effects were not performed. All tests were two-sided, and P < 0.05 was considered statistically significant.

## Results

### Study selection and study characteristics

The PRISMA flow diagram summarizes the study-selection process (**Figure 1**). The database search identified 1,742 records from PubMed (n = 345), Scopus (n = 383), Web of Science Core Collection (n = 380), Embase (n = 612), and the Cochrane Library (n = 22). After 695 duplicates were removed, 1,047 records underwent title and abstract screening. At this stage, 163 records were excluded, including non-English publications, reviews, conference abstracts or proceedings, and case reports. Full texts were retrieved for 884 reports and assessed for eligibility; 836 were excluded because they involved an ineligible population, intervention, comparator, study design, outcome, or non-breast-conserving surgical setting. Forty-eight comparative studies met the eligibility criteria and were included in the systematic review and quantitative synthesis (13, 14, 17–62).

**Figure 1:**
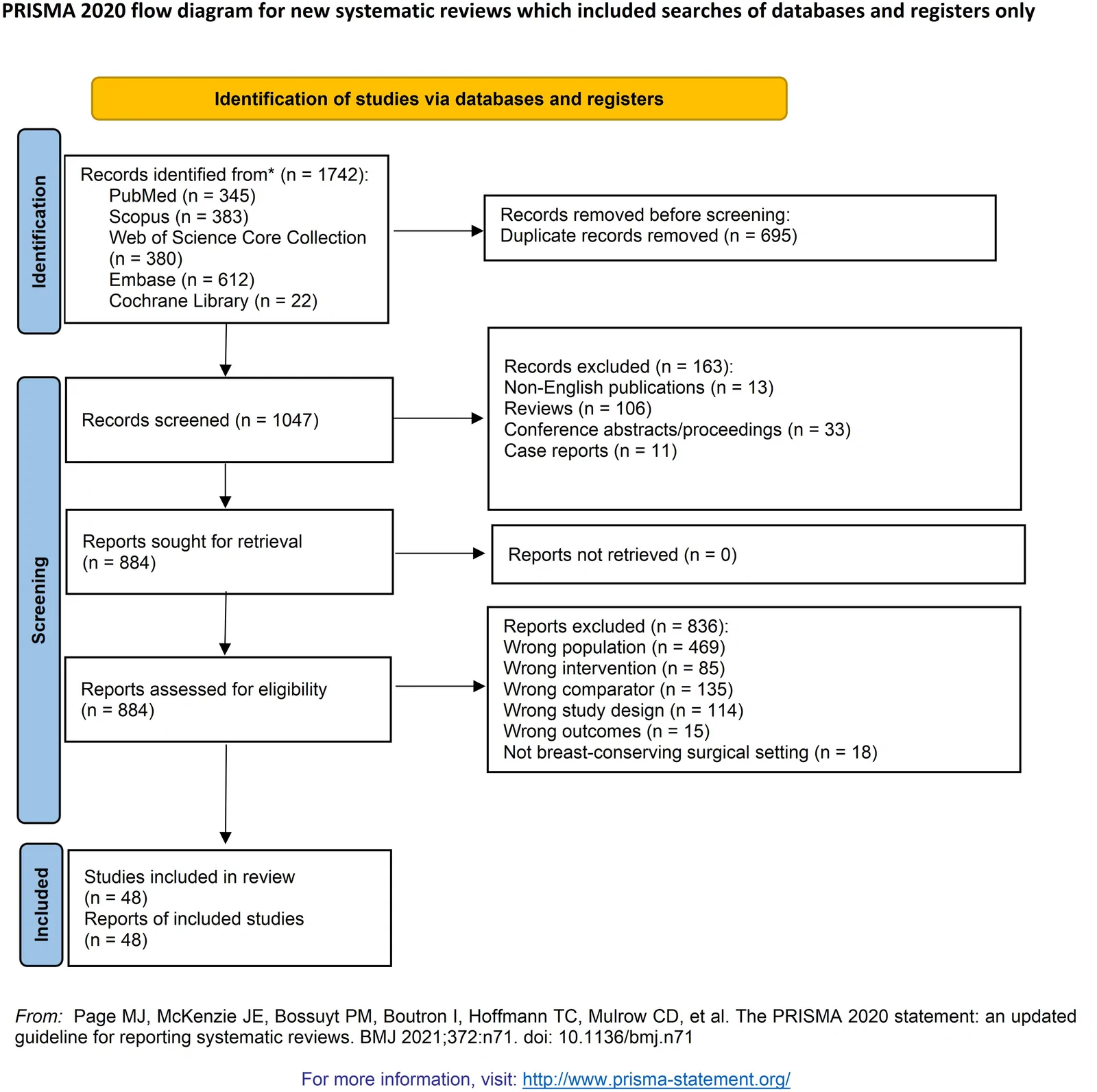
PRISMA flow diagram of the study-selection process. *From: Page, M.J., McKenzie, J.E., Bossuyt, P.M., Boutron, I., Hofmann, T.C., Mulrow, C.D., Shamseer, L., Tetzlaff, J.M., Akl, E.A., Brennan, S.E., et al. (2021). The PRISMA 2020 statement: an updated guideline for reporting systematic reviews. Syst Rev. Mar 29;10(1):89. doi: 10.1186/s13643-021-01626-4. PMID: 33781348; PMCID: PMC8008539.

The included studies were published between 2005 and 2026 and represented a broad range of healthcare settings across Asia, Europe, North America, South America, and the Middle East. Most were retrospective comparative cohort studies. The evidence base also included prospective comparative studies (40, 42), matched case-control or propensity score-matched studies (14, 46, 52, 59), registry or administrative database studies (14, 21, 44, 56), and one randomized controlled trial (55).

The 48 studies comprised 124,903 breast-conserving surgery observations, including 28,085 in the OBCS group and 96,818 in the conventional BCS group. Because the original reports did not use a uniform analytical unit, denominators were retained according to the unit reported in each study, including patient-, breast-, procedure-, or tumor-level denominators. Study populations included invasive breast cancer, ductal carcinoma *in situ*, mixed invasive and *in situ* disease, early-stage breast cancer, locally advanced disease after neoadjuvant therapy, and broader BCS cohorts.

The definition and technical scope of OBCS varied across studies. Reported techniques included level I or II oncoplastic procedures, volume displacement, volume replacement, therapeutic mammoplasty or mammaplasty, oncoplastic reduction, glandular reshaping, local flap reconstruction, perforator flap-based reconstruction, and other immediate reshaping procedures performed within a breast-conserving framework. Comparator groups included conventional BCS, standard lumpectomy, wide local excision, partial mastectomy, segmentectomy, or quadrantectomy without immediate oncoplastic reconstruction.

The number of studies contributing to each meta-analysis varied because margin definitions, reoperation categories, follow-up duration, and complication reporting were not uniform. Initial positive surgical margin was reported in 19 comparisons, re-excision in 29 comparisons, completion/conversion mastectomy in 20 comparisons, and any margin-related reoperation in 27 comparisons. Secondary outcomes included close margin, negative/clear margin, local recurrence or ipsilateral breast tumor recurrence, locoregional recurrence, and overall complications. Key study characteristics are summarized in **Table 1**. Detailed baseline and surgical characteristics are provided in **Supplementary Table 1**, and detailed margin-related, oncologic, safety, and patient-reported outcomes are provided in **Supplementary Table 2**.

**Table 1:** Study characteristics and key margin-related outcomes of included comparative studies.

Study	Country/ Region	Study design	Population/ stage	OBCS type	Comparator	Total n	OBCS n	C-BCS n	FU (mo)	PM, OBCS n/N (%)	PM, C-BCS n/N (%)	RE, OBCS n/N (%)	RE, C-BCS n/N (%)	CM, OBCS n/N (%)	CM, C-BCS n/N (%)	Main outcome(s) for synthesis
Abdelkader et al. (17)	Egypt	Single-center, retrospective comparative study	Primary invasive BC/DCIS	Mixed VD/VR OBCS	Conventional BCS	310	155	155	NR	NR	NR	12/155 (7.7)†	30/155 (19.4)†	0/155 (0)	NR	Margin width; reshaving†; LR; complications; cosmesis
Abidi et al. (18)	Pakistan	Retrospective cohort study	Early/LABC breast cancer	Mixed VD/VR OBCS	Conventional BCS	422†	249	173	NR	5/249 (2.0)†	31/173 (17.9)†	2/249 (0.8)†	17/173 (9.8)	0/249 (0)	7/173 (4.0)	PM; RE; CM
Almeida et al. (19)	Brazil	Single-center, retrospective cohort study	Breast cancer treated with BCS	Mammaplasty or locoregional flap	Non-oncoplastic BCS	866	98	768	50.4	6/97 (6.2)	78/759 (10.3)	5/98 (5.1)	98/768 (12.7)	4/98 (4.1)	33/768 (4.3)	PM; RE; CM; LR; complications; time to RT
Almojel et al. (20)	Saudi Arabia	Single-center, retrospective cohort study	Breast cancer, stages 0–IV	OBCS with immediate reconstruction	Standard BCS	237	137	100	20.4 (8.4–36.0)	24/137 (17.5)	5/100 (5.0)	36/137 (26.3)	13/100 (13.0)	NR	NR	PM; RE; LR; complications
André et al. (21)	Sweden	Multicenter, register-based retrospective cohort study	Primary invasive BC; T1–T3; BCS+RT	Simple/complex OPS	Standard BCS	4178	458	3720	64 (24–110)	NR	NR	27/458 (5.9)	197/3720 (5.3)	NR	NR	RE; margin width; LR; OS/BCSS
Atallah et al. (22)	Lebanon	Single-center, retrospective case-control study	Invasive/*in situ* breast cancer; primary surgery	Mainly level II VD OBCS	Conventional BCS	277	193	84	66 (24–108)/72 (24–120)	NR	NR	9/193 (4.7)	6/84 (7.1)	NR	NR	RE; margin width; LR
Azhar et al. (23)	Indonesia	Multicenter, retrospective cohort study	Stage I–III breast cancer	Mixed VD/VR OPS	Conventional BCS	82	39	43	30 d	1/39 (2.5)	4/43 (9.3)	4/39 (10.3)†	17/43 (39.5)†	NR	NR	PM; RE†; complications; LR
Bali et al. (24)	United Kingdom	Single-center, retrospective cohort study	Invasive carcinoma/DCIS treated with BCS	Mammoplasty or chest-wall perforator flap	WLE	201	35	166	NR	2/35 (5.7)	26/166 (15.7)	6/35 (17.1)	36/166 (21.7)	0/35 (0)	9/166 (5.4)	PM; RE; CM
Bargon et al. (13)	Netherlands	Prospective multicenter longitudinal cohort study	Invasive breast cancer/DCIS treated with BCS	VD/VR OP-BCS	Conventional BCS	1918	290	1628	24	38/290 (13.1)	245/1628 (15.0)	14/290 (4.8)	86/1628 (5.3)	2/290 (0.7)	7/1628 (0.4)	PM; RE/CM; time to RT; PROM
Behluli et al. (25)	Germany/Switzerland	Multicenter, retrospective comparative study	Primary nonmetastatic invasive/in situ/mixed BC	Level II OBS/mammoplasty techniques	Conventional BCS	343	52	291	NR	NR	NR	4/52 (7.7)	100/291 (34.4)	0/52 (0)	33/291 (11.3)	RE; CM; margin width; complications
Caixeiro et al. (26)	Portugal	Single-center, retrospective cohort study	Breast cancer; BCT/ORM; pTis–pT2	Oncoplastic reduction mammoplasty	BCT/standard tumor resection	215	90	125	89.8 (3–158)	8/87 (9.2)	31/121 (25.6)	0/90 (0)	4/125 (3.2)	5/90 (5.6)	22/125 (17.5)	PM; RE; CM; any MRR; margin width; recurrence
Calì Cassi et al. (27)	Italy	Single-center, retrospective observational cohort study	Breast cancer treated with lumpectomy	Therapeutic mammoplasty/tissue transfer	Non-oncoplastic BCS	215 procedures†	61	154	44.8/43.3	NR	NR	4/61 (6.5)	20/154 (12.9)	NR	NR	RE; LR; complications; time to RT
Caziuc et al. (28)	Romania	Single-center, retrospective comparative study	DCIS or invasive BC with DCIS component	Level I/II OBCS; mainly lateral mammoplasty	Simple lumpectomy	76	29	47	NR	0/29 (0)	5/47 (10.6)	0/29 (0)	5/47 (10.6)	0/29 (0)	2/47 (4.3)	PM; RE; CM; margin width; complications
Çelik et al. (29)	Türkiye	Single-center, retrospective cohort study	Breast cancer with DCIS component	Level II OBS	Conventional BCS	163	40	123	24 (18–36)	NR	NR	3/40 (10.0)	37/123 (30.1)	NR	NR	RE; LR; BREAST-Q PROM
Chakravorty et al. (30)	United Kingdom	Single-center, retrospective comparative cohort study	Breast cancer treated with BCS	Therapeutic mammoplasty/Grisotti/Benelli oBCS	Standard BCS	590	150	440	28 (6–81)	NR	NR	4/150 (2.7)	59/440 (13.4)	6/150 (4.0)	5/440 (1.1)	RE; CM; LR; distant relapse
Chauhan et al. (31)	India	Single-center, prospective comparative study with historical controls	Early breast cancer; T1/T2, N0/N1	Mixed VD/VR OBS	Conventional BCS	79	33	46	18 (6–30)/38 (12–64)	0/33 (0)	5/46 (10.9)	0/33 (0)	2/46 (4.3)	0/33 (0)	3/46 (6.5)	PM; close margin; RE; CM; LRR; complications
Chauhan et al. (32)	India	Single-center, prospective comparative study with historical controls	LABC after NACT; cT3/T4, N0–N2	Mixed VD/VR OBS	Conventional BCS	100	57	43	18 (6–30)/34 (14–44)	1/57 (1.8)	3/43 (7.0)	0/57 (0)	1/43 (2.3)	1/57 (1.8)	2/43 (4.7)	PM; close margin; RE; CM; LRR; complications
Crown et al. (33)	United States	Single-center, retrospective before-after cohort study	Invasive/noninvasive breast cancer; BCS subset for RE	Institution-wide oncoplastic BCS program	Standard surgery/standard BCS	610†	329	281	NR	NR	NR	60/329 (18.2)†	90/281 (32.0)†	6/329 (1.8)†	5/281 (1.8)†	RE†; CM†
Çulcu et al. (34)	Türkiye	Single-center, retrospective cohort study	Invasive breast cancer; pure DCIS and NACT excluded	Level II volume-displacement OPS	Conventional lumpectomy	460	237	223	52	NR	NR	26/237 (11.0)†	40/223 (17.9)†	NR	NR	MRR/reoperation†; margin width; LR; complications
De Lorenzi et al. (35)	Italy	Single-center, retrospective cohort study	Pure DCIS treated with conservation + RT	Oncoplastic conservation + RT	Conservation alone + RT	419	44	375	92.4/110.4	2/44 (4.5)	13/374 (3.5)	NA†	NA†	NA†	NA†	PM; close margin; IBTR; DFS/OS
Di Micco et al. (36)	United Kingdom	Single-center, retrospective cohort study	Large-breasted women with early (<3 cm) unilateral BC	Bilateral reduction mammoplasty/therapeutic mammoplasty	Standard unilateral BCS/WLE	157	70	87	36 (9.8–76)	3/70 (4.3)†	14/87 (16.1)†	2/70 (2.9)	14/87 (16.1)	NR	NR	PM†; RE; complications; time to RT; PROM
Down et al. (37)	United Kingdom	Single-center, retrospective cohort study	Early invasive BC or DCIS requiring BCS	Therapeutic mammoplasty/local dermoglandular flaps	Standard WLE	158 procedures†	37	121†	23.7 overall; 29.3/22.1	NR	NR	1/37 (2.7)	18/121 (14.9)	1/37 (2.7)	17/121 (14.0)	Margin width; RE; CM; LR
Falade et al. (38)	United States	Single-center, retrospective cohort study	Stage I–III invasive lobular carcinoma	Immediate oncoplastic closure or ORM	Standard lumpectomy	494	168	326	49.2/55.2 *vs* 118.8†	54/167 (32.3)†	132/319 (41.4)†	37/168 (22.0)†	69/326 (21.2)†	15/168 (8.9)	85/326 (26.1)	PM; RE†; CM; successful BCS; RFS
Garreffa et al. (39)	United Kingdom	Single-center, retrospective cohort study	Invasive or *in situ* BC treated with BCS	Parenchymal displacement or chest-wall perforator flap OBS	Simple WLE	123 breasts†	63 breasts†	60	NR	NR	NR	6/63 (9.5)	12/60 (20.0)	2/63 (3.2)	4/60 (6.7)	RE; CM; any MRR; specimen dimensions
Giacalone et al. (40)	France	Prospective comparative study	Breast cancer, tumor diameter ≥15 mm; eligible for either approach	Round-block or inverted-T oncoplastic mammaplasty	Standard quadrantectomy	74	31	43	NR	3/31 (9.7)	7/43 (16.3)	0/31 (0)	1/43 (2.3)	4/31 (12.9)	7/43 (16.3)	PM; close margin; RE; CM; margin width
Gulcelik et al. (41)	Türkiye	Single-center, prospective comparative study	Invasive breast cancer; stages I–IIIA	Bilateral reduction mammoplasty	Standard quadrantectomy	268	106	162	33 (16–88)/37 (20–124)	9/106 (8.5)	18/162 (11.1)	11/106 (10.4)	16/162 (9.9)†	0/106 (0)	8/162 (4.9)	PM; RE; CM; LR
Heeg et al. (14)	Denmark	Nationwide population-based cohort study with PSM	Invasive breast cancer without distant metastasis	OPS: volume displacement/reduction/replacement	BCS without OPS	18188	5003	13185	2 mo†	NR	NR	706/5003 (14.1)†	2057/13185 (15.6)†	NR (3.2)	NR (3.7)	RE/MRR; CM
Kaur et al. (42)	Italy	Prospective nonrandomized comparative study	Breast cancer; pTis/T1/T2	Oncoplastic surgery; mostly volume displacement	Standard quadrantectomy	60	30	30	NR	1/30 (3.3)	1/30 (3.3)	NR	NR	NR	NR	PM; close/negative margin
Kelemen et al. (43)	Hungary	Single-center, retrospective cohort study	Stage 0–III breast cancer	Volume displacement OBCS	Conventional BCS	700	350	350	51/52	NR	NR	19/350 (5.4)	38/350 (10.9)	9/350 (2.6)	20/350 (5.7)	RE; CM; MRR; LR/LRR
Kilmer et al. (44)	United States	National claims database retrospective cohort study	Breast cancer treated with lumpectomy ± same-day reduction	Oncoplastic breast reduction surgery	Lumpectomy alone	30268	15134	15134	12 mo†	NR	NR	1129/15134 (7.5)	1662/15134 (11.0)	826/15134 (5.5)	814/15134 (5.4)	RE; CM; MRR; complications
Lima et al. (45)	Brazil	Multicenter, retrospective real-world cohort study	Stage 0–III breast cancer; BCS + adjuvant RT	Therapeutic mammoplasty; volume displacement O-BCS	Standard BCS	685	135	550	42	2/113 (1.8)	23/503 (4.6)	2/135 (1.5)†	22/550 (4.0)†	NR	NR	PM; re-operative surgery; LRR/RFS/OS
Lim et al. (46)	Singapore	Matched case-control study	Histologically proven breast cancer treated with BCS	Round block oncoplastic technique; level II volume displacement	Standard WLE	22	11	11	24 (11–29)/50 (10–60)	NR	NR	1/11 (9.1)	2/11 (18.2)	1/11 (9.1)	1/11 (9.1)	RE; CM; LRR; complications/cosmesis
Lopez Rios et al. (47)	Canada	Single-center, retrospective cohort study	Breast cancer; stage IV/metastatic/recurrent excluded	OBCS level I/II; tissue rearrangement/mammoplasty/local flap	Lumpectomy without reconstruction	1290	307	983	55.7/66.5	32/307 (10.4)	99/983 (10.1)	NR	NR	NR	NR	PM; LRR; complications; time to RT; DFS
Mukhtar et al. (48)	United States	Single-center, retrospective cohort study	Stage I–III invasive lobular carcinoma; attempted BCS	Lumpectomy with level 1 or level 2 OPS	Lumpectomy alone	365 tumors†	88	277	NR	30/88 (34.1)	127/277 (45.8)	23/88 (26.1)†	75/277 (27.1)†	12/88 (13.6)	81/277 (29.2)	PM; RE; CM; breast conservation
Nanda et al. (49)	United Kingdom	Single-center, retrospective cohort study	Breast cancer treated with BCS; WLE or VR-OPS	Volume replacement OPS; LICAP/LTAP lateral chest wall perforator flaps	Standard WLE	159	79	80	89.5/87	7/79 (8.9)†	21/80 (26.3)†	5/79 (6.3)	4/80 (5.0)	2/79 (2.5)	9/80 (11.3)	PM/clear margin; RE; CM; LR; complications
Niinikoski et al. (50)	Finland	Single-center, retrospective cohort study	Primary invasive BC/DCIS; BCS	Mixed level I/II OBCS	Conventional BCS	1800	611	1189	75 (2–94)	NR	NR	17/611 (2.8)	42/1189 (3.5)	39/611 (6.4)	54/1189 (4.5)	RE; CM; any MRR; LR
Nisiri et al. (51)	Iran	Single-center, comparative retrospective study	Stage I–III unilateral breast cancer	Mixed oncoplastic techniques	Conventional BCS	317	154	163	24	16/154 (10.4)	30/163 (18.4)	5/154 (3.2)†	10/163 (6.1)†	8/154 (5.2)†	12/163 (7.4)†	PM; RE; CM
Oh et al. (52)	South Korea	Single-center, retrospective cohort study with PSM	Early breast cancer; BCS+RT	Level II OPS; VD/VR techniques	Conventional BCS	742	371	371	84.4 ± 22.7/87.9 ± 24.8	26/371 (7.0)†	28/371 (7.5)†	19/371 (5.1)	12/371 (3.2)	NR	NR	Close/involved margin; RE; IBTRFS; RFS
Rose et al. (53)	Denmark	Population-based retrospective cohort study	Primary invasive breast cancer; BCS	Level I/II OBS	Conventional BCS	1596	197	1399	49.2/67.2†	19/190 (10.0)†	218/1364 (16.0)†	NR	NR	NR	NR	Nonradical primary excision; time to adjuvant therapy; DFS; OS
Sagiroglu et al. (54)	Türkiye	Single-center, retrospective comparative study	Unilateral early BC; stage I–IIB	Level I/II OBS	Non-oncoplastic BCS	288	138	150	74 (7–89)	9/138 (6.5)†	12/150 (8.0)†	9/138 (6.5)†	12/150 (8.0)†	NR	NR	Frozen-section PM/RE; LR; DFS; complications
Saha et al. (55)	India	Randomized controlled trial	Early breast cancer; tumor ≤4 cm	Volume-displacement oncoplasty	Simple closure	104	52	52	54 (12–70)	NR	NR	9/52 (17.3)	1/52 (1.9)	NR	NR	RE; any MRR; complications; cosmesis
Shah et al. (56)	United States	National claims database retrospective cohort study	Adult female breast cancer; BCT + RT	Oncoplastic reduction with lumpectomy	Lumpectomy alone	53165	1552	51613	≥6 mo	NR	NR	NR	NR	NR	NR	Any MRR†; complications; time to RT
Tenofsky et al. (57)	United States	Single-surgeon, retrospective cohort study	Female breast cancer treated with lumpectomy	Adjacent tissue transfer/donut mastopexy/therapeutic mammoplasty	Non-oncoplastic lumpectomy	142	58	84	24.6 ± 10.2/26.2 ± 16.5	NR	NR	3/58 (5.2)	11/84 (13.1)	NR	NR	RE; complications; time to RT; cosmesis
Tokui et al. (58)	Japan	Single-center, retrospective cohort study	Stage 0–III breast cancer; no neoadjuvant therapy	BCS with immediate LDMF reconstruction	BCS alone	1185	145	1040	61 (1–104)	6/145 (4.1)	112/1040 (10.8)	2/145 (1.4)	36/1040 (3.5)	0/145 (0)	16/1040 (1.5)	PM; RE; CM; final PM; LRFS
Vieira et al. (59)	Brazil	Matched case-control study	LABC after NACT; BCS	Mixed oncoplastic techniques	Classic BCS/quadrantectomy	78	26	52	67.1	0/26 (0)†	0/52 (0)†	0/26 (0)†	0/52 (0)†	0/26 (0)†	0/52 (0)†	PM; margin width; LR; LRR; survival
Vohra et al. (60)	Pakistan	Multicenter, retrospective cohort study	Stage I–III breast cancer; BCS	Mixed OPS; volume displacement/replacement	Standard BCS	481	277	204	1 (30 d)	6/277 (2.2)	32/204 (15.7)	0/277 (0)	17/204 (8.3)	4/277 (1.4)	NR	PM; RE; CM; any MRR; complications
Wijesinghe et al. (61)	Sri Lanka	Two-center, retrospective cohort study	Early/LABC, non-metastatic breast cancer; BCS	Level I/II O-BCS; volume displacement/replacement	Standard BCS	127	73	54	12	NR	NR	1/73 (1.4)	9/54 (16.7)	NR	NR	RE; margin width; complications; PROM
Zhou et al. (62)	China	Single-center, retrospective comparative cohort study	Stage I–II breast cancer; BCS	Volume displacement OPBS	Conventional BCS	106	43	63	2 (0.25–6)	2/43 (4.65)	11/63 (17.46)	1/43 (2.33)†	0/63 (0)†	NR	1/63 (1.59)	PM; any MRR; short-term safety; cosmesis

Data are shown as n/N (%) unless otherwise indicated. Detailed margin definitions are reported in **Supplementary Table 2**. ^†^Non-standard, derived, incompletely separated, or internally inconsistent outcome/denominator; these data were handled in sensitivity analyses when included in pooling. ‡ Definition differs from the pure category or represents a composite category as reported in the original study. BC, breast cancer; BCS, breast-conserving surgery; BCSS, breast cancer-specific survival; C-BCS, conventional breast-conserving surgery; CM, completion/conversion mastectomy; DCIS, ductal carcinoma *in situ*; DFS, disease-free survival; FU, follow-up; IBTR, ipsilateral breast tumor recurrence; LABC, locally advanced breast cancer; LR, local recurrence; MRR, margin-related reoperation; NA, not applicable; NACT, neoadjuvant chemotherapy; NR, not reported; OS, overall survival; OBCS, oncoplastic breast-conserving surgery; PROM, patient-reported outcome measure; PM, positive margin; RE, re-excision; RFS, recurrence-free survival; RT, radiotherapy; VD, volume displacement; VR, volume replacement; WLE, wide local excision.

### Methodological quality

Among the 47 non-randomized studies assessed using the Newcastle–Ottawa Scale, scores ranged from 6 to 9. Five studies scored 9, 20 scored 8, 19 scored 7, and three scored 6, indicating overall moderate-to-high methodological quality. The randomized trial (55) was assessed separately and was not included in the Newcastle–Ottawa Scale score distribution because this tool is not intended for randomized evidence. Quality assessment results are summarized in **Table 2**.

**Table 2:** Methodological quality and risk-of-bias assessment of the included studies.

Study	Country	Study design	The Newcastle-Ottawa Scale (NOS)			
			Selection	Comparability	Outcome	Total score
Abdelkader et al. (17)	Egypt	Single-center, retrospective comparative study	* * * *	*	* *	7
Abidi et al. (18)	Pakistan	Retrospective cohort study	* * * *	*	* *	7
Almeida et al. (19)	Brazil	Single-center, retrospective cohort study	* * * *	* *	* * *	9
Almojel et al. (20)	Saudi Arabia	Single-center, retrospective cohort study	* * * *	* *	* *	8
André et al. (21)	Sweden	Multicenter, register-based retrospective cohort study	* * * *	* *	* * *	9
Atallah et al. (22)	Lebanon	Single-center, retrospective case-control study	* * *	*	* * *	7
Azhar et al. (23)	Indonesia	Single-center, retrospective cohort study	* * *	*	* *	6
Bali et al. (24)	United Kingdom	Single-center, retrospective cohort study	* * *	*	* *	6
Bargon et al. (13)	Netherlands	Multicenter, longitudinal cohort study	* * * *	* *	* * *	9
Behluli et al. (25)	Germany/Switzerland	Multicenter, retrospective comparative study	* * * *	*	* *	7
Caixeiro et al. (26)	Portugal	Single-center, retrospective cohort study	* * * *	*	* * *	8
Calì Cassi et al. (27)	Italy	Single-center, retrospective observational cohort study	* * * *	*	* *	7
Caziuc et al. (28)	Romania	Single-center, retrospective comparative study	* * * *	*	* *	7
Çelik et al. (29)	Türkiye	Single-center, retrospective cohort study	* * * *	*	* * *	8
Chakravorty et al. (30)	United Kingdom	Single-center, retrospective comparative cohort study	* * * *	*	* * *	8
Chauhan et al. (31)	India	Single-center, prospective comparative study with historical controls	* * * *	*	* *	7
Chauhan et al. (32)	India	Single-center, prospective nonrandomized comparative study with retrospective/historical controls	* * * *	*	* *	7
Crown et al. (33)	United States	Single-center, retrospective comparative cohort study	* * * *	*	* *	7
Çulcu et al. (34)	Türkiye	Single-center, retrospective cohort study	* * * *	*	* * *	8
De Lorenzi et al. (35)	Italy	Single-center, retrospective cohort study	* * * *	*	* * *	8
Di Micco et al. (36)	United Kingdom	Single-center, retrospective cohort study	* * * *	*	* *	7
Down et al. (37)	United Kingdom	Single-center, retrospective cohort study	* * * *	*	* *	7
Falade et al. (38)	United States	Single-center, retrospective cohort study	* * * *	* *	* *	8
Garreffa et al. (39)	United Kingdom	Single-center, retrospective cohort study	* * *	*	* * *	7
Giacalone et al. (40)	France	Prospective comparative study	* * * *	*	* *	7
Gulcelik et al. (41)	Türkiye	Single-center, retrospective comparative study	* * * *	*	* *	7
Heeg et al. (14)	Denmark	Nationwide population-based cohort study with PSM	* * * *	* *	* * *	9
Kaur et al. (42)	Italy	Prospective nonrandomized comparative study	* * * *	*	* *	7
Kelemen et al. (43)	Hungary	Single-center, retrospective cohort study	* * * *	*	* * *	8
Kilmer et al. (44)	United States	National claims database retrospective cohort study	* * * *	* *	* *	8
Lima et al. (45)	Brazil	Multicenter, retrospective real-world cohort study	* * * *	* *	* *	8
Lim et al. (46)	Singapore	Matched case-control study	* * * *	* *	* *	8
Lopez Rios et al. (47)	Canada	Single-center, retrospective cohort study	* * * *	* *	* *	8
Mukhtar et al. (48)	United States	Single-center, retrospective cohort study	* * * *	* *	* *	8
Nanda et al. (49)	United Kingdom	Single-center, retrospective cohort study	* * * *	*	* * *	8
Niinikoski et al. (50)	Finland	Single-center, retrospective cohort study	* * * *	*	* * *	8
Nisiri et al. (51)	Iran	Single-center, comparative retrospective study	* * * *	*	* *	7
Oh et al. (52)	South Korea	Single-center, retrospective cohort study with PSM	* * * *	* *	* * *	9
Rose et al. (53)	Denmark	Single-center, retrospective cohort study	* * * *	* *	* *	8
Sagiroglu et al. (54)	Türkiye	Single-center, retrospective comparative study	* * * *	*	* *	7
Saha et al. (55)	India	Randomized controlled trial	NA	NA	NA	NA
Shah et al. (56)	United States	National claims database retrospective cohort study	* * * *	* *	* *	8
Tenofsky et al. (57)	United States	Single-surgeon, retrospective cohort study	* * *	*	* *	6
Tokui et al. (58)	Japan	Single-center, retrospective cohort study	* * * *	* *	* *	8
Vieira et al. (59)	Brazil	Matched case-control study	* * * *	* *	* *	8
Vohra et al. (60)	Pakistan	Multicenter, retrospective cohort study	* * * *	*	* *	7
Wijesinghe et al. (61)	Sri Lanka	Single-center, retrospective cohort study	* * * *	*	* *	7
Zhou et al. (62)	China	Single-center, retrospective comparative cohort study	* * * *	*	* * *	8

*1 point; maximum NOS score = 9 points. Non-randomized comparative studies were assessed using the Newcastle–Ottawa Scale (NOS) across the cohort-study domains of Selection, Comparability, and Outcome. In the Comparability domain, one star indicates adjustment or matching for the most important prognostic confounder(s), whereas two stars indicate additional adjustment or matching for other relevant clinicopathologic confounders. The randomized controlled trial by Saha et al. was not eligible for NOS assessment and was assessed separately using RoB 2. Higher NOS scores indicate higher methodological quality. BCS, breast-conserving surgery; LDMF, latissimus dorsi myocutaneous flap; NOS, Newcastle–Ottawa Scale; OBCS, oncoplastic breast-conserving surgery; OPS, oncoplastic surgery; PSM, propensity score matching; RCT, randomized controlled trial; RoB 2, revised Cochrane risk-of-bias tool for randomized trials; WLE, wide local excision. Each star represents one point awarded according to the Newcastle–Ottawa Scale (NOS) criteria.

### Primary margin-related outcomes

#### Initial positive surgical margin

Nineteen comparisons contributed data to the primary meta-analysis of initial positive surgical margin (13, 19, 20, 23, 24, 26, 28, 31, 32, 35, 40–42, 45, 47, 51, 58, 60, 62). Random-effects pooling showed that OBCS was associated with a significantly lower risk of an initial positive margin than conventional BCS (159/2,054 *vs*. 748/6,518; RR 0.57, 95% CI 0.40–0.80; P = 0.001; **Figure 2**). Moderate-to-substantial heterogeneity was observed (Tau² = 0.27; Chi² = 46.16, df = 18, P = 0.0003; I² = 61%).

**Figure 2:**
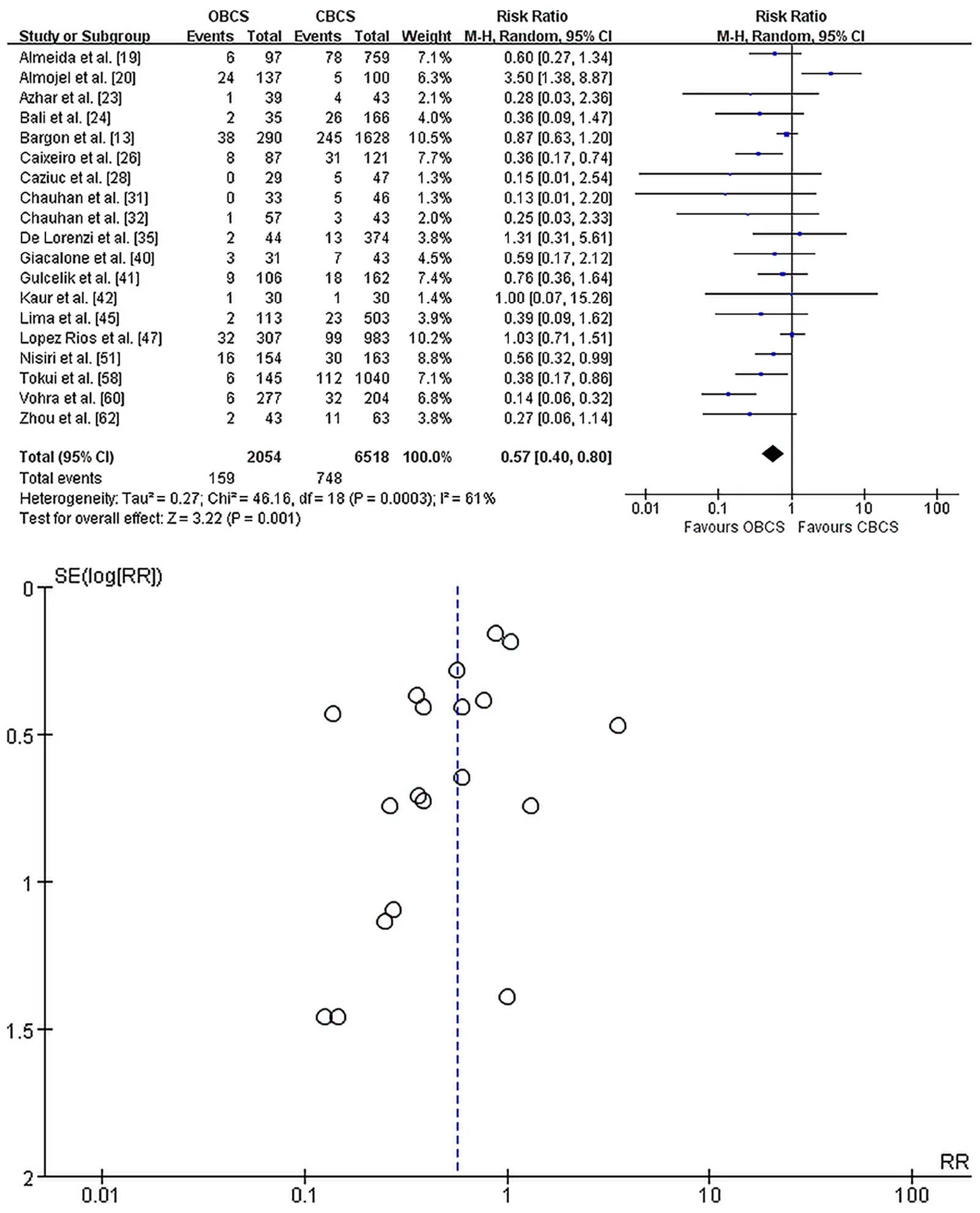
Forest and funnel plots for initial positive surgical margin after OBCS versus conventional BCS. The upper panel shows the random-effects Mantel–Haenszel forest plot, and the lower panel shows the corresponding funnel plot. Effect estimates are risk ratios comparing OBCS with conventional BCS.

To address potential overlap between the cohorts reported by Vohra et al. and Abidi et al. (18, 60), a sensitivity analysis replaced the Vohra dataset with the Abidi dataset. The pooled estimate was nearly identical (158/2,026 vs. 747/6,487; RR 0.56, 95% CI 0.40–0.80; P = 0.001; **Supplementary Figure 1**), with similar heterogeneity (Tau² = 0.28; Chi² = 47.44, df = 18, P = 0.0002; I² = 62%).

#### Re-excision

Twenty-nine comparisons reported re-excision outcomes (13, 19–22, 24–32, 36, 37, 39, 40, 43, 44, 46, 49, 50, 52, 55, 57, 58, 60, 61). OBCS was associated with a significantly lower risk of re-excision than conventional BCS (1,324/19,085 *vs*. 2,536/26,615; RR 0.59, 95% CI 0.46–0.77; P < 0.0001; **Figure 3**). Heterogeneity was moderate (Tau² = 0.18; Chi² = 70.88, df = 28, P < 0.0001; I² = 60%).

**Figure 3:**
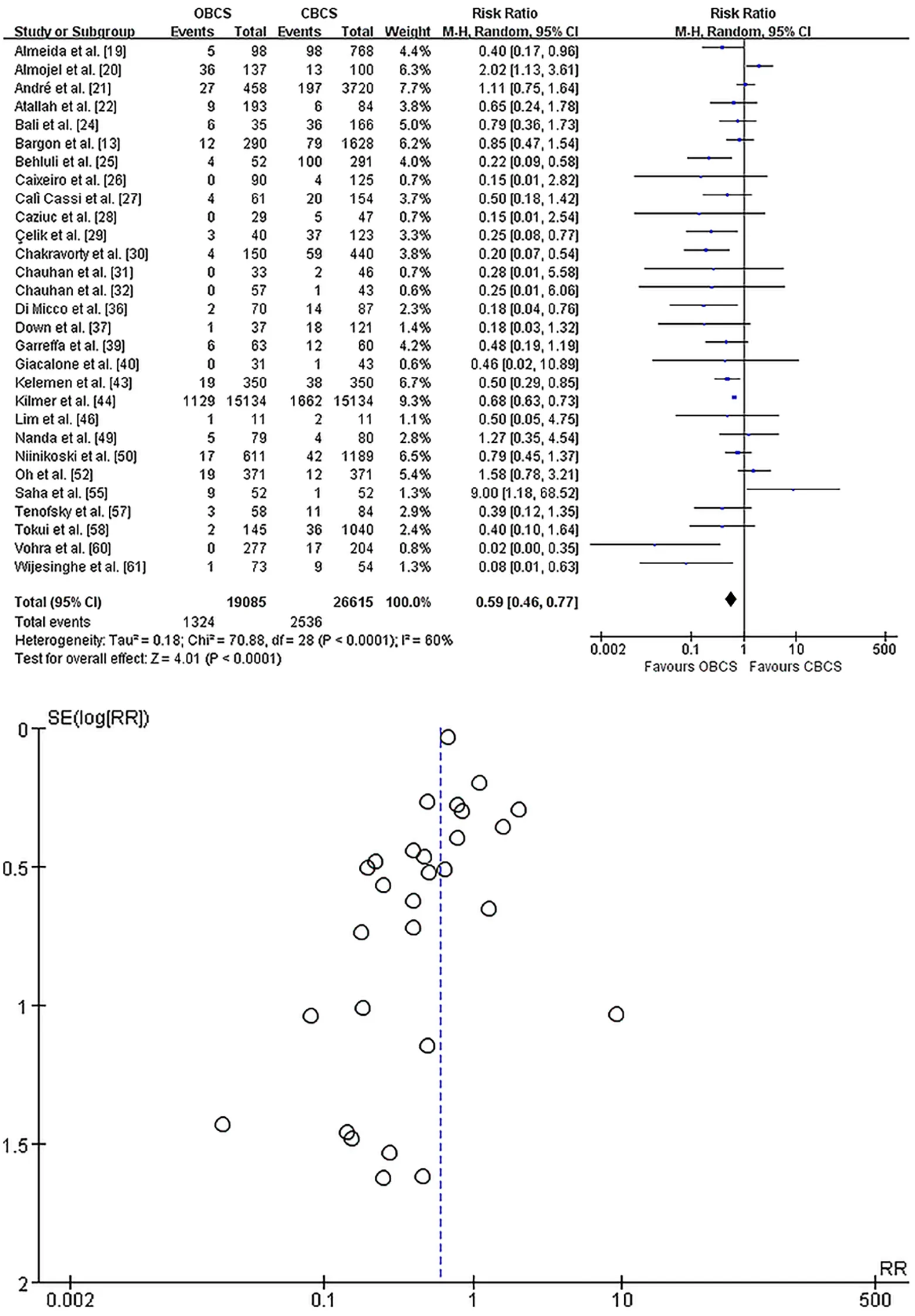
Forest and funnel plots for re-excision after OBCS versus conventional BCS. The upper panel shows the random-effects Mantel–Haenszel forest plot, and the lower panel shows the corresponding funnel plot.

In the overlapping-cohort sensitivity analysis, the Vohra dataset was replaced with the Abidi dataset (18, 60). The pooled association remained essentially unchanged (1,326/19,057 *vs*. 2,536/26,584; RR 0.58, 95% CI 0.45–0.75; P < 0.0001; **Supplementary Figure 2**), with comparable heterogeneity (Tau² = 0.19; Chi² = 73.04, df = 28, P < 0.00001; I² = 62%).

#### Completion/conversion mastectomy

Twenty comparisons contributed to the meta-analysis of completion/conversion mastectomy (13, 19, 24–26, 28, 30–32, 37–41, 43, 44, 46, 49, 50, 58). OBCS was associated with a significantly lower risk of completion/conversion mastectomy than conventional BCS (917/17,569 *vs*. 1,151/22,070; RR 0.62, 95% CI 0.43–0.90; P = 0.01; **Figure 4**). Moderate-to-substantial heterogeneity was observed (Tau² = 0.26; Chi² = 51.83, df = 19, P < 0.0001; I² = 63%).

**Figure 4:**
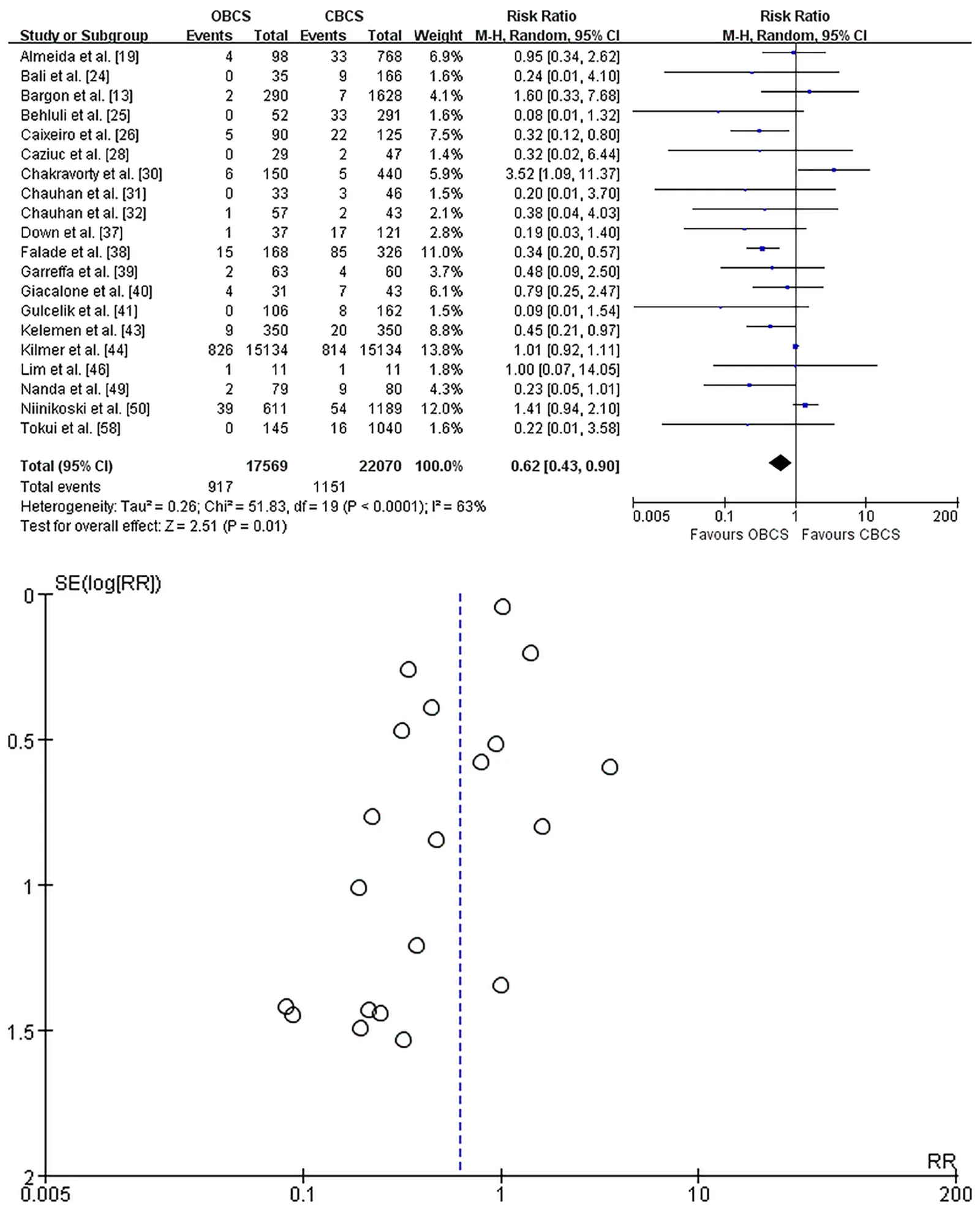
Forest and funnel plots for completion/conversion mastectomy after OBCS versus conventional BCS. The upper panel shows the random-effects Mantel–Haenszel forest plot, and the lower panel shows the corresponding funnel plot.

Two sensitivity analyses were performed for this outcome. First, studies with definition or data-coding caveats were added (33, 51). The pooled estimate remained statistically significant and similar in magnitude (931/18,052 *vs*. 1,168/22,514; RR 0.65, 95% CI 0.46–0.91; P = 0.01; **Supplementary Figure 3**), with similar heterogeneity (Tau² = 0.22; Chi² = 52.32, df = 21, P = 0.0002; I² = 60%). Second, the Falade dataset was replaced with the Mukhtar dataset to address potential cohort overlap (38, 48). The association also remained significant (914/17,489 *vs*. 1,147/22,021; RR 0.66, 95% CI 0.47–0.94; P = 0.02; **Supplementary Figure 4**), with slightly lower heterogeneity (Tau² = 0.19; Chi² = 42.70, df = 19, P = 0.001; I² = 56%).

#### Any margin-related reoperation

Twenty-seven comparisons contributed to the analysis of any margin-related reoperation (13, 19–22, 24–32, 36, 37, 39–41, 43, 44, 49, 50, 52, 57, 61, 62). OBCS was associated with a significantly lower risk of any margin-related reoperation than conventional BCS (2,110/18,749 *vs*. 3,340/25,533; RR 0.63, 95% CI 0.51–0.78; P < 0.0001; **Figure 5**). Moderate-to-substantial heterogeneity was observed (Tau² = 0.12; Chi² = 70.18, df = 26, P < 0.00001; I² = 63%).

**Figure 5:**
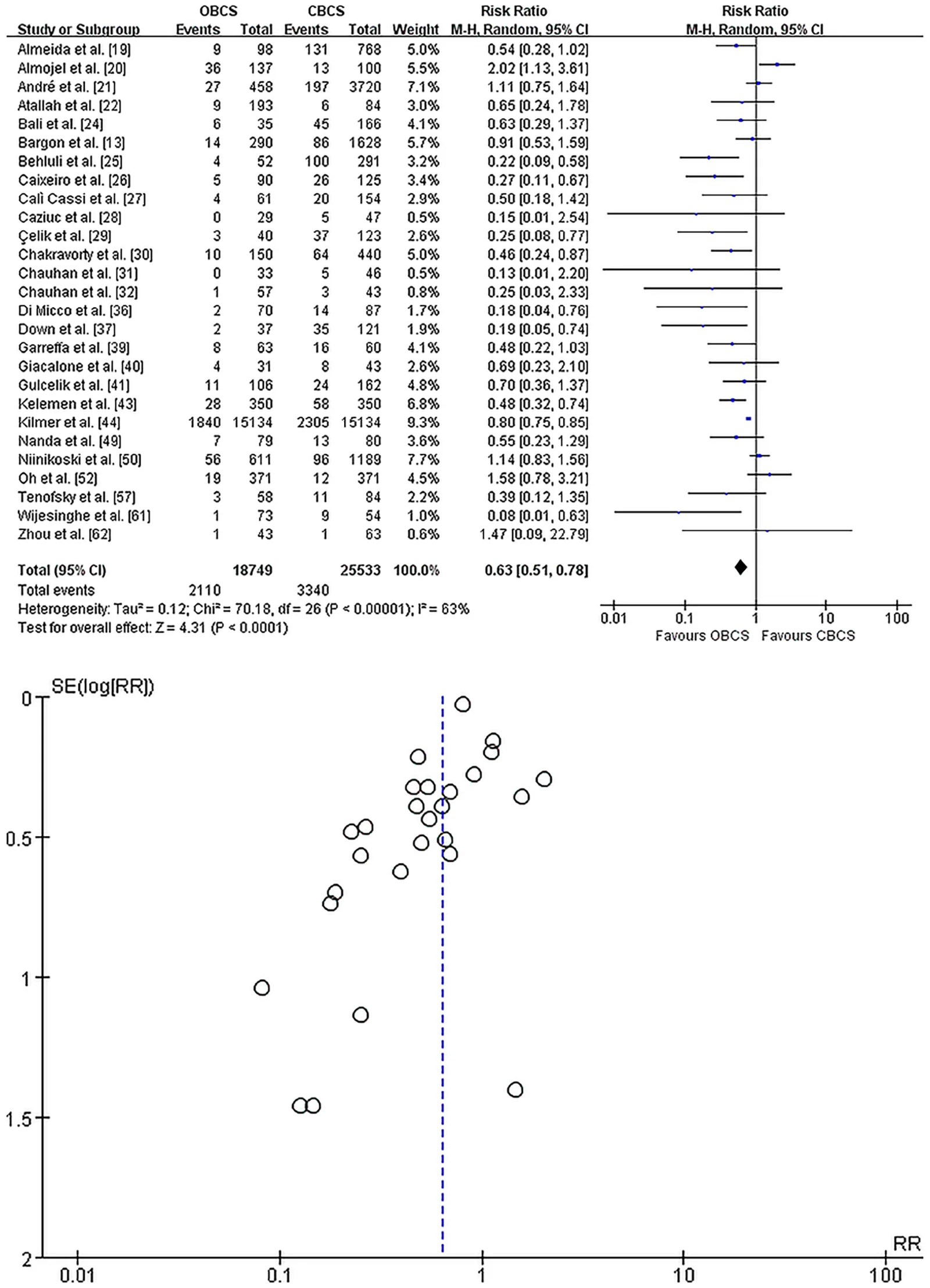
Forest and funnel plots for any margin-related reoperation after OBCS versus conventional BCS. The upper panel shows the random-effects Mantel–Haenszel forest plot, and the lower panel shows the corresponding funnel plot.

Because this composite endpoint included different forms of margin-related reoperation and could be influenced by coding-based ascertainment, a sensitivity analysis excluded two large registry or administrative database studies (21, 44). The pooled estimate remained statistically significant (243/3,157 *vs*. 838/6,679; RR 0.55, 95% CI 0.41–0.73; P < 0.0001; **Supplementary Figure 5**), and heterogeneity remained similar (Tau² = 0.27; Chi² = 64.92, df = 24, P < 0.0001; I² = 63%).

### Secondary margin-status outcomes

#### Close margin

Six comparisons contributed to the primary meta-analysis of close margin (31, 32, 35, 40–42). The pooled estimate showed no statistically significant difference between OBCS and conventional BCS (25/301 *vs*. 103/698; RR 0.67, 95% CI 0.39–1.16; P = 0.16; **Figure 6**). Low-to-moderate heterogeneity was observed (Tau² = 0.15; Chi² = 7.45, df = 5, P = 0.19; I² = 33%).

**Figure 6:**
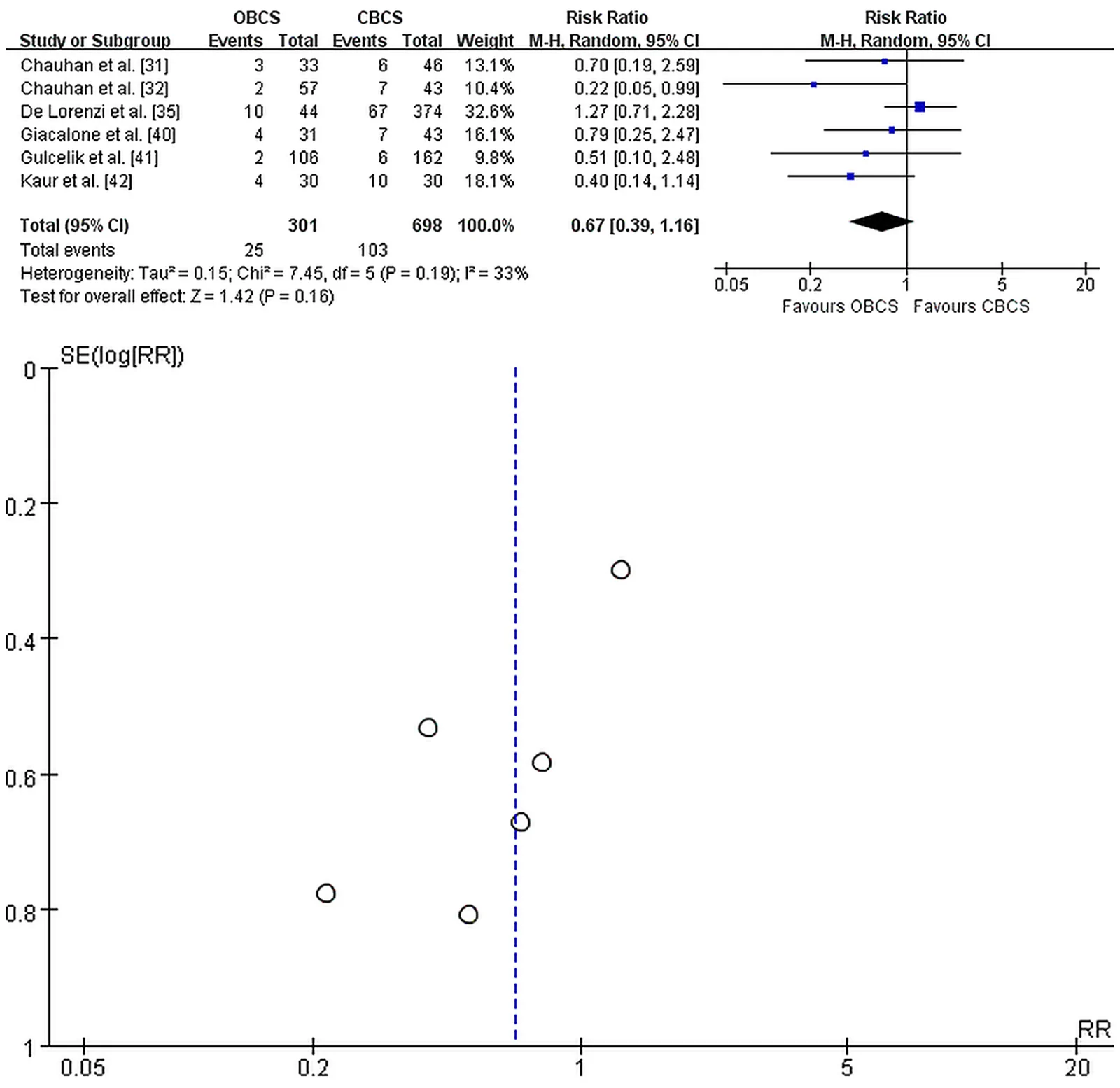
Forest and funnel plots for close margin after OBCS versus conventional BCS. The upper panel shows the random-effects Mantel–Haenszel forest plot, and the lower panel shows the corresponding funnel plot.

Several additional studies reported close margins using broader or less directly comparable definitions, including close/involved margins or inadequate-margin thresholds (13, 37, 51, 52, 57). When these studies were added, the estimate remained in the same direction but did not reach conventional statistical significance (95/1,211 *vs*. 372/3,065; RR 0.74, 95% CI 0.55–1.01; P = 0.06; **Supplementary Figure 6**), with similar heterogeneity (Tau² = 0.07; Chi² = 14.31, df = 10, P = 0.16; I² = 30%).

#### Negative/clear margin

Nineteen comparisons reported negative or clear margin outcomes (13, 19 ,20 ,23, 24, 26, 28, 31, 32, 35, 40, 41, 45, 47, 49, 51, 58, 60, 62). Because a negative/clear margin is a favorable endpoint, an RR greater than 1 favored OBCS. Random-effects pooling showed that OBCS was associated with a higher likelihood of achieving negative/clear margins than conventional BCS (1,924/2,103 *vs*. 5,757/6,568; RR 1.07, 95% CI 1.04–1.11; P < 0.0001; **Figure 7**). Substantial heterogeneity was observed (Tau² = 0.00; Chi² = 62.64, df = 18, P < 0.00001; I² = 71%).

**Figure 7:**
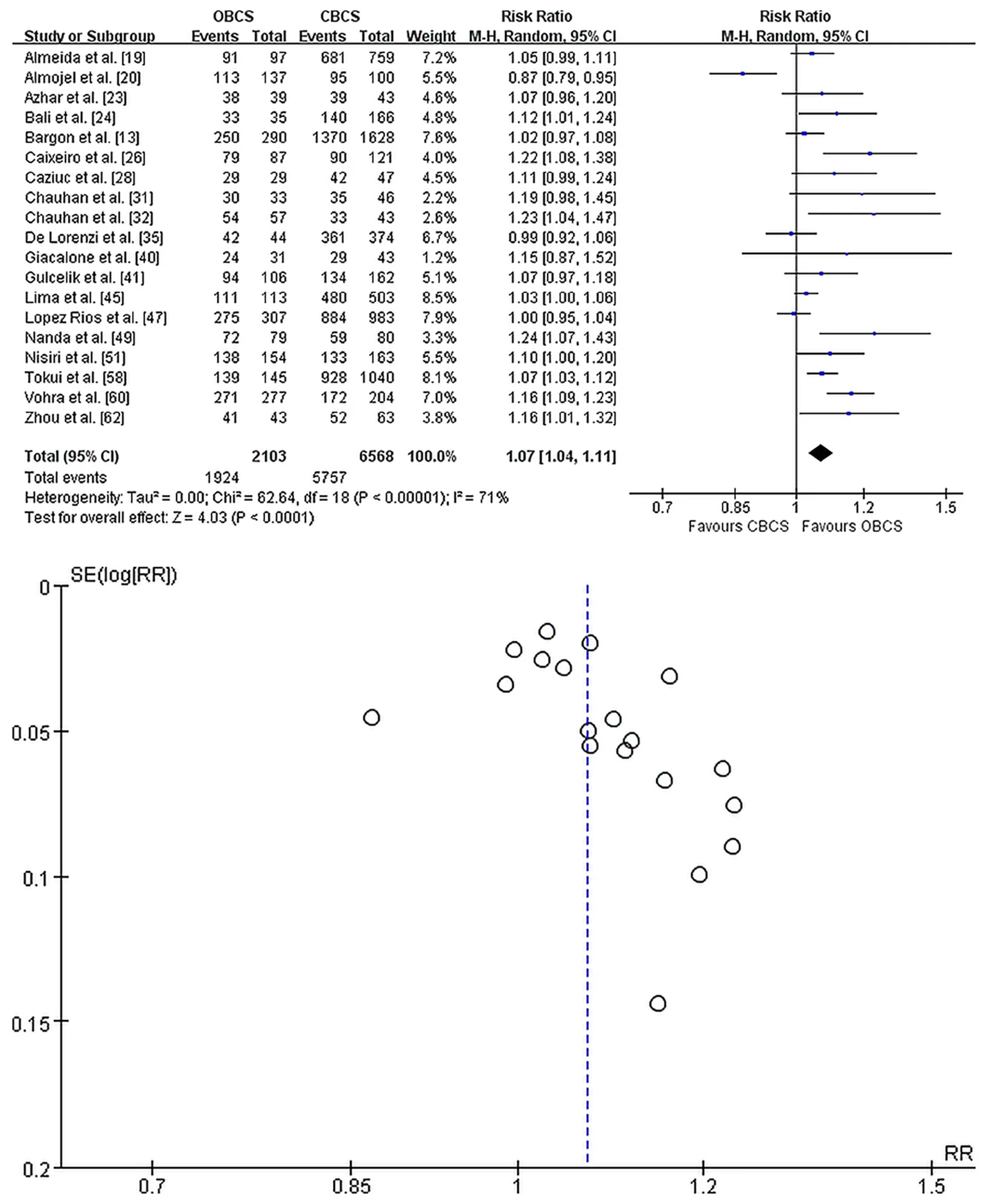
Forest and funnel plots for negative/clear margin after OBCS versus conventional BCS. The upper panel shows the random-effects Mantel–Haenszel forest plot, and the lower panel shows the corresponding funnel plot. For this favorable outcome, an RR greater than 1 favors OBCS.

Replacing the potentially overlapping Vohra dataset with the Abidi dataset (18, 60) did not materially change the magnitude or statistical significance of the pooled estimate (1,897/2,075 *vs*. 5,727/6,537; RR 1.07, 95% CI 1.04–1.11; P < 0.0001; **Supplementary Figure 7**). Heterogeneity remained substantial (Tau² = 0.00; Chi² = 65.23, df = 18, P < 0.00001; I² = 72%).

A second sensitivity analysis added studies reporting negative or clear margins using broader, non-standard, or less directly comparable definitions (36, 37, 42, 52, 53, 59). To avoid combining separate sensitivity assumptions, this analysis retained the Vohra dataset and did not include Abidi, Falade, or Mukhtar (18, 38, 48, 60). The pooled estimate remained statistically significant (2,593/2,827 *vs*. 7,474/8,593; RR 1.07, 95% CI 1.04–1.11; P < 0.0001; **Supplementary Figure 8**), and heterogeneity remained substantial (Tau² = 0.00; Chi² = 89.75, df = 24, P < 0.00001; I² = 73%).

### Oncologic and surgical safety outcomes

#### Local recurrence/ipsilateral breast tumor recurrence

Eighteen comparisons reported local recurrence or ipsilateral breast tumor recurrence (17, 19–23, 27, 29, 30, 34, 35, 41, 43, 49, 50, 54, 57, 59). Random-effects pooling showed no statistically significant difference between OBCS and conventional BCS (65/2,839 *vs*. 244/7,996; RR 0.84, 95% CI 0.64–1.11; P = 0.22; **Figure 8**). No statistical heterogeneity was observed (Tau² = 0.00; Chi² = 16.27, df = 17, P = 0.50; I² = 0%).

**Figure 8:**
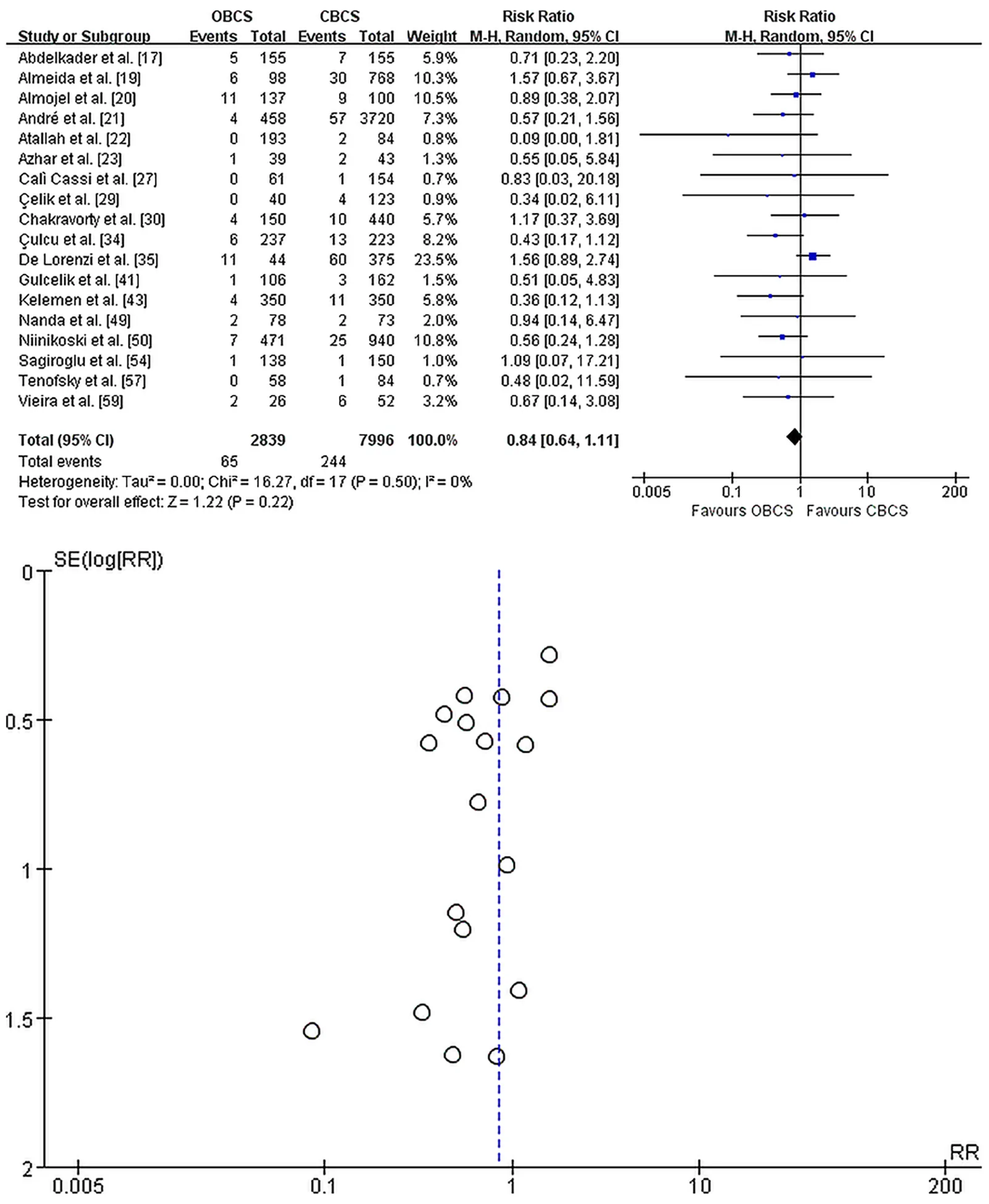
Forest and funnel plots for local recurrence or ipsilateral breast tumor recurrence after OBCS versus conventional BCS. The upper panel shows the random-effects Mantel–Haenszel forest plot, and the lower panel shows the corresponding funnel plot.

#### Locoregional recurrence

Ten comparisons contributed to the meta-analysis of locoregional recurrence (20, 31, 32, 35, 43, 45–47, 50, 59). The pooled estimate showed no statistically significant difference between OBCS and conventional BCS (36/1,571 *vs*. 113/3,448; RR 0.75, 95% CI 0.51–1.11; P = 0.15; **Figure 9**). No statistical heterogeneity was observed (Tau² = 0.00; Chi² = 8.32, df = 9, P = 0.50; I² = 0%).

**Figure 9:**
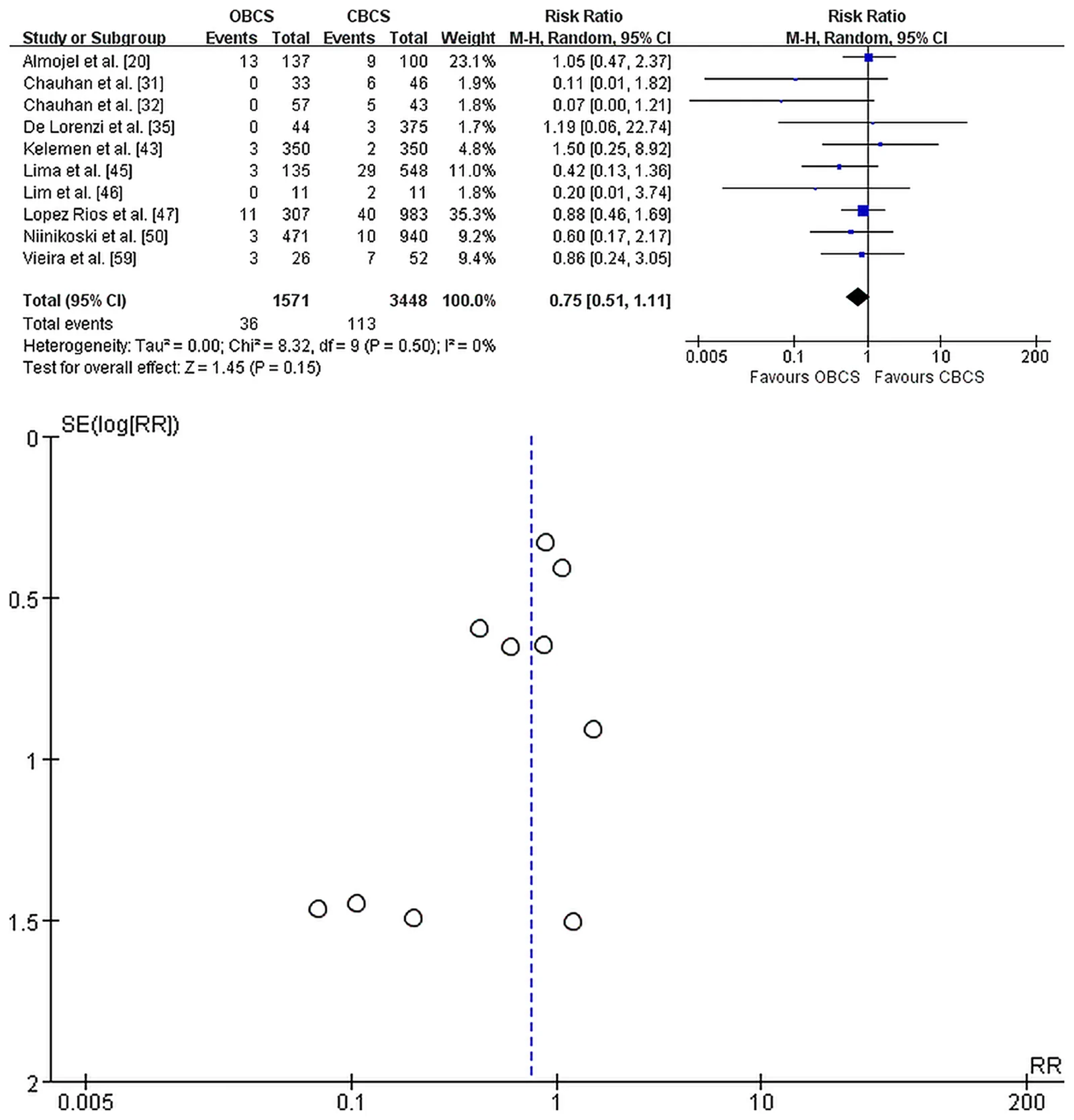
Forest and funnel plots for locoregional recurrence after OBCS versus conventional BCS. The upper panel shows the random-effects Mantel–Haenszel forest plot, and the lower panel shows the corresponding funnel plot.

### Overall complications

Sixteen comparisons reported overall complications (19, 20, 23, 25, 27, 28, 31, 32, 36, 43, 44, 49, 52, 56, 60, 61). Definitions and severity reporting varied across studies; available complication types and severity classifications, including Clavien–Dindo grading when reported, are summarized in **Supplementary Table 2**. Because severity-specific data were not reported consistently, complications could not be pooled by severity grade. Complication laterality was also inconsistently reported. Almeida et al. (19) explicitly included complications involving both the index breast and the symmetrized contralateral breast, whereas most other studies did not provide sufficient detail to distinguish ipsilateral from contralateral events. In the primary random-effects analysis, OBCS was associated with a higher risk of overall complications than conventional BCS (4,769/18,412 *vs*. 10,580/69,385; RR 1.40, 95% CI 1.02–1.92; P = 0.04; **Figure 10**). Heterogeneity was substantial (Tau² = 0.22; Chi² = 301.40, df = 15, P < 0.00001; I² = 95%).

**Figure 10:**
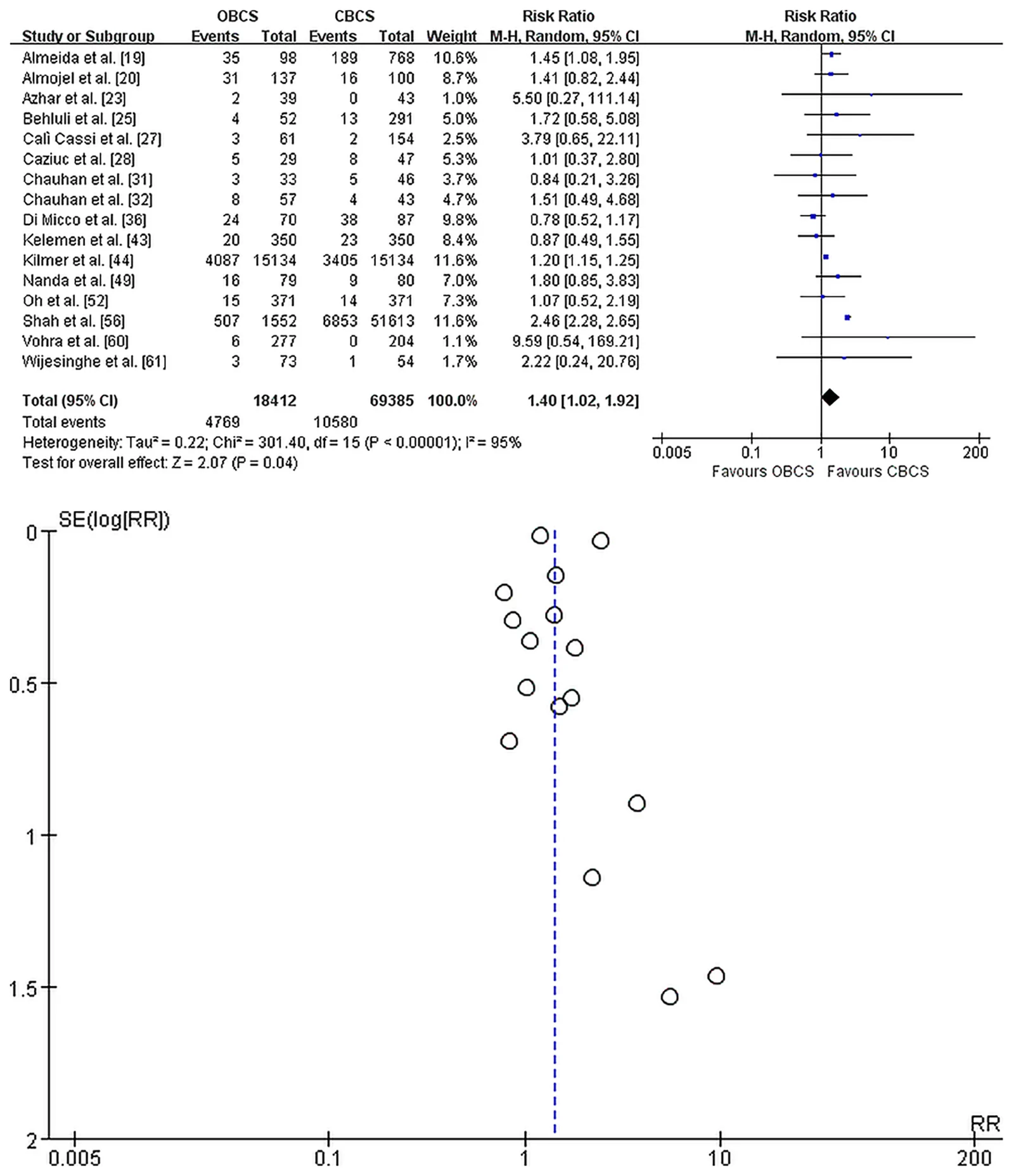
Forest and funnel plots for overall complications after OBCS versus conventional BCS. The upper panel shows the random-effects Mantel–Haenszel forest plot, and the lower panel shows the corresponding funnel plot.

Because complication definitions, severity reporting, and ascertainment methods varied across studies, and because two large registry or administrative database studies may have differed from institutional cohorts in how complications were coded and captured (44, 56), these studies were excluded in a sensitivity analysis. The pooled estimate was attenuated to borderline statistical significance (175/1,726 *vs*. 322/2,638; RR 1.23, 95% CI 1.00–1.50; P = 0.05; **Supplementary Figure 9**). Heterogeneity decreased markedly (Tau² = 0.02; Chi² = 14.48, df = 13, P = 0.34; I² = 10%). All sensitivity analyses are summarized in **Supplementary Table 3**.

### Small-study effects

Funnel plots were visually inspected alongside the corresponding forest plots for the primary and sensitivity analyses. Because several endpoints included fewer than 10 comparisons and multiple outcomes showed moderate-to-substantial heterogeneity, the funnel plots were considered exploratory. Formal tests for small-study effects were not performed. Accordingly, the funnel plots should not be interpreted as definitive evidence for or against publication bias.

## Discussion

In this systematic review and meta-analysis of 48 comparative studies comprising 124,903 BCS observations, OBCS was associated with lower risks of initial positive surgical margin, re-excision, completion/conversion mastectomy, and any margin-related reoperation, as well as a higher likelihood of negative/clear margins. Close-margin findings were less definitive, and no significant differences were observed in local or locoregional recurrence. Overall complication risk was higher in the primary analysis, but this association was attenuated after exclusion of registry or administrative-database cohorts. These findings suggest that OBCS may improve the margin-to-reoperation pathway in appropriately selected patients, although interpretation is limited by predominantly observational evidence and heterogeneity in surgical technique, outcome definitions, and complication ascertainment.

The margin findings are clinically plausible. Consensus guidelines define adequate margins as “no ink on tumor” for invasive cancer and 2 mm for DCIS treated with whole-breast irradiation (4, 5), but these pathological thresholds do not eliminate the intraoperative trade-off between oncologic clearance and cosmetic preservation. The randomized cavity-shave trial showed that margin outcomes can be modified by operative strategy (8). OBCS extends this principle by combining excision with immediate volume displacement or replacement; the American Society of Breast Surgeons classifies level 1 displacement as generally <20% and level 2 as approximately 20–50% of breast volume (63). In our analysis, OBCS reduced the risk of an initial positive margin by 43% (RR 0.57, 95% CI 0.40–0.80) and modestly increased the likelihood of achieving a negative/clear margin (RR 1.07, 95% CI 1.04–1.11). The smaller relative effect for negative margins is expected because negative margins were already common in both groups, whereas positive margins are less frequent but clinically consequential. Because specimen volume, planned margin width, tumor-to-breast volume ratio, and cavity-shave use were inconsistently reported, these findings should not be interpreted as evidence that all OBCS procedures remove more tissue. Rather, OBCS may allow a larger oncologic resection in selected patients while preserving breast form.

Close margin warrants separate interpretation because it is threshold-dependent rather than a uniform pathological state. Included studies used <1 mm, <2 mm, close/involved, or broader “inadequate margin” definitions, and invasive cancer and DCIS are governed by different thresholds. In a cohort of 1,491 patients with DCIS, Tadros et al. found no significant difference in 10-year locoregional recurrence between margins <2 mm and ≥2 mm among patients receiving radiotherapy (4.8% *vs*. 3.3%; HR 0.8, 95% CI 0.2–3.2), whereas the same distinction was strongly prognostic without radiotherapy (30.9% *vs*. 5.4%; HR 5.5, 95% CI 1.8–16.8) (64). A meta-analysis of invasive breast cancer likewise emphasized the importance of separating close margins from tumor-on-ink margins (65). Consistent with this heterogeneity, our primary close-margin estimate favored OBCS but was not statistically significant (RR 0.67, 95% CI 0.39–1.16); the estimate remained similar when broader definitions were added (RR 0.74, 95% CI 0.55–1.01). Thus, this endpoint appears particularly sensitive to pathological thresholds, DCIS burden, radiotherapy use, and institutional re-excision policies.

The clinical importance of improved margin control is most apparent in the need for additional surgery. Re-excision entails repeat anesthesia, wound healing, pathology review, and potential delay in adjuvant treatment. In a claims-based analysis of 9,837 women, 23.2% underwent another breast operation, with a mean interval of 24 days and an additional mean cost of $16,072 (66); more recent data reported reoperation rates of 21.1% in commercially insured patients and 14.9% in Medicare patients, with substantial incremental costs (7). In our analysis, OBCS was associated with a lower risk of re-excision (RR 0.59, 95% CI 0.46–0.77), but re-excision is not a direct surrogate for positive margin because decisions may also reflect close margins, DCIS extent, residual calcifications, multifocality, institutional policy, and patient preference. Completion/conversion mastectomy is a more consequential endpoint because it represents unplanned abandonment of the breast-conserving strategy rather than a primary mastectomy selected during initial treatment planning. OBCS was associated with a lower risk of completion/conversion mastectomy (RR 0.62, 95% CI 0.43–0.90). The association remained statistically significant when studies with definition or coding caveats were added (RR 0.65, 95% CI 0.46–0.91) and when a potentially overlapping cohort was replaced (RR 0.66, 95% CI 0.47–0.94). This finding was also consistent with the nationwide Danish study (14). The composite endpoint of any margin-related reoperation should likewise be interpreted alongside its components because re-excision and completion/conversion mastectomy differ in severity and clinical meaning, and composite outcomes can obscure such differences (67). The composite estimate (RR 0.63, 95% CI 0.51–0.78) was consistent in direction with the separate re-excision and mastectomy analyses and remained significant after exclusion of two large registry or administrative studies (RR 0.55, 95% CI 0.41–0.73). Overall, these findings support a lower likelihood of requiring a margin-related second operation after OBCS, while preserving the distinction between re-excision with continued breast conservation and conversion to mastectomy.

Recurrence provides an important oncologic safety endpoint. Local recurrence/ipsilateral breast tumor recurrence (RR 0.84, 95% CI 0.64–1.11) and locoregional recurrence (RR 0.75, 95% CI 0.51–1.11) did not differ significantly between groups, with no statistical heterogeneity. These findings are broadly consistent with contemporary OBCS cohorts and comparative analyses (12, 13, 21, 52, 68) and are clinically relevant because tissue rearrangement and fewer reoperations could raise concerns about occult residual disease or compromised local control. However, recurrence is a downstream endpoint influenced by radiotherapy, tumor biology, systemic therapy, margin policy, and follow-up duration. In the EORTC boost trial, the 20-year cumulative incidence of ipsilateral breast tumor recurrence was 16.4% without a boost and 12.0% with a boost (69). After oncoplastic reconstruction, tumor-bed displacement may also complicate boost planning and interpretation of close or positive margins (70). Because these factors also influence recurrence, similar recurrence rates cannot be attributed to the index operation alone. The recurrence findings are therefore reassuring but not definitive. They do not establish long-term oncologic superiority, a survival benefit, or equivalence across all tumor subtypes, OBCS techniques, and adjuvant treatment settings.

The complication findings temper the margin-related benefits. Depending on technique, OBCS involves additional tissue mobilization or reconstruction and may increase wound-healing problems, seroma, hematoma, infection, fat necrosis, skin-flap compromise, or nipple–areolar complex ischemia (71, 72). In the primary analysis, overall complication risk was higher with OBCS (RR 1.40, 95% CI 1.02–1.92; I² = 95%), but after exclusion of two registry or administrative-database studies, the estimate was attenuated to borderline statistical significance (RR 1.23, 95% CI 1.00–1.50; P = 0.05) and I² fell to 10%. This marked change indicates sensitivity to study design and ascertainment. Administrative data may identify events through billing, procedure, readmission, or diagnosis codes, whereas institutional cohorts may rely on chart review, prospective recording, or surgeon-defined categories; methodological guidance emphasizes transparent reporting of coding algorithms and outcome definitions (73). Moreover, overall complication rates do not necessarily reflect major morbidity when severity grading is inconsistent. Hillberg et al. reported complications in 37.5% of 150 OBCS patients, but radiotherapy was delayed because of complications in only 8.2% (74); Moen et al. reported an overall complication rate of 31.2% but a major complication rate of 3.9% (75). The observed increase in overall complications should therefore be interpreted as a potential clinical trade-off rather than as evidence that the margin-related benefits are unimportant. This distinction is especially relevant because the pooled “overall complication” endpoint does not represent a uniform measure of major postoperative morbidity.

These findings support selective rather than routine use of OBCS. Patients most likely to benefit are those for whom conventional lumpectomy is technically or cosmetically challenging, including patients with a high tumor-to-breast volume ratio, larger or more extensive DCIS components, selected multifocal disease still suitable for conservation, lesions in cosmetically sensitive locations, and macromastia or ptosis suitable for therapeutic mammoplasty. Good-practice guidance emphasizes multidisciplinary selection based on patient-, tumor-, and treatment-specific factors (76). Preoperative imaging should define disease extent; localization should correspond to the planned resection; the margin strategy and specimen orientation should be explicit; and the anticipated reshaping should be communicated to pathology and radiation oncology because tissue rearrangement can alter the postoperative tumor bed. When boost irradiation may be required, consensus guidance recommends clip placement along the cavity walls and adequate operative documentation (77). Accordingly, OBCS is best considered a planned multidisciplinary approach rather than a routine extension of breast-conserving surgery.

This review differs from previous reviews (10, 68, 78, 79), which largely evaluated the overall efficacy, safety, and aesthetic rationale of OBCS, by focusing on the sequence from margin status to subsequent surgery. We separately evaluated initial positive margin, close margin, negative/clear margin, re-excision, completion/conversion mastectomy, and any margin-related reoperation. This approach distinguishes pathological margin status from its subsequent surgical consequences and directly addresses whether OBCS is associated with better initial margin control and fewer margin-driven reoperations. The distinction matters because avoiding mastectomy at the initial operation is not equivalent to maintaining breast conservation without additional surgery.

Future studies should standardize both OBCS classification and outcome reporting. Prospective studies and harmonized registries should distinguish level I from level II procedures and volume displacement from volume replacement, while capturing tumor-to-breast volume ratio, disease extent, localization method, specimen volume, and planned margin strategy. Positive, close, and negative margins should be reported separately with explicit thresholds for invasive cancer and DCIS; re-excision and completion/conversion mastectomy should be separated and accompanied by reasons for additional surgery; complications should be graded by severity; time to radiotherapy and systemic therapy should be captured; and patient-reported outcomes should be assessed with validated instruments such as the BREAST-Q breast-conserving therapy module (76, 80). Randomized trials may be difficult because anatomy, tumor location, cosmetic preference, surgeon expertise, and equipoise influence treatment choice. High-quality prospective multicenter cohorts or registries with harmonized definitions, prespecified comparative questions, and adjustment for confounding by indication may therefore offer a more feasible approach to strengthening the evidence base.

This study has several strengths. Its clinically focused design separated the margin-to-reoperation pathway into distinct endpoints, and the 48 comparative studies provided a large evidence base for these analyses. Positive margin, close margin, negative/clear margin, re-excision, completion/conversion mastectomy, and any margin-related reoperation were analyzed separately whenever definitions permitted. Sensitivity analyses also addressed overlapping cohorts, non-standard definitions, coding caveats, and large registry or administrative datasets.

Several limitations should also be acknowledged. Most studies were observational and retrospective, leaving the possibility of residual selection bias and confounding by indication. OBCS techniques were heterogeneous, and most studies did not permit technique-specific comparisons. Margin definitions, close-margin thresholds, indications for reoperation, complication definitions and severity grading, and analytical units varied across studies. Complication laterality was also incompletely reported, preventing consistent separation of ipsilateral OBCS complications from events related to contralateral symmetrization. Some pooled analyses therefore combined patient-, breast-, procedure-, or tumor-level observations according to the analytical unit used in the original study. Interpretation of recurrence was limited by heterogeneous follow-up and incomplete reporting of radiotherapy, systemic therapy, tumor biology, and pathological risk factors. Complication estimates were particularly sensitive to study design and ascertainment method. Cosmetic outcomes, quality of life, time to adjuvant therapy, cost, and patient-reported outcomes were insufficiently reported for robust quantitative synthesis, and certainty of evidence was not formally graded using GRADE. Accordingly, the findings should be interpreted as associations rather than evidence of causal superiority.

In conclusion, OBCS was associated with more favorable margin control and fewer margin-related reoperations than conventional BCS, without a detectable increase in local or locoregional recurrence during the available follow-up. These benefits should be balanced against uncertainty in complication risk because complication reporting was heterogeneous. In appropriately selected patients, OBCS may improve the durability of breast conservation, but prospective studies using standardized technique classification, margin definitions, reasons for reoperation, complication grading, adjuvant treatment timing, and patient-reported outcomes are needed to identify the patients and procedures most likely to benefit.

## Data Availability

The original contributions presented in the study are included in the article/**Supplementary Material**. Further inquiries can be directed to the corresponding author.

## Supplementary material

Supplementary Figure 1 Sensitivity analysis for initial positive surgical margin after replacing the potentially overlapping Vohra dataset with the Abidi dataset. The upper panel shows the random-effects Mantel–Haenszel forest plot, and the lower panel shows the corresponding funnel plot.

Supplementary Figure 2 Sensitivity analysis for re-excision after replacing the potentially overlapping Vohra dataset with the Abidi dataset. The upper panel shows the random-effects Mantel–Haenszel forest plot, and the lower panel shows the corresponding funnel plot.

Supplementary Figure 3 Sensitivity analysis for completion/conversion mastectomy after adding studies with definition or data-coding caveats. The upper panel shows the random-effects Mantel–Haenszel forest plot, and the lower panel shows the corresponding funnel plot.

Supplementary Figure 4 Sensitivity analysis for completion/conversion mastectomy after replacing the Falade dataset with the Mukhtar dataset to address potential cohort overlap. The upper panel shows the random-effects Mantel–Haenszel forest plot, and the lower panel shows the corresponding funnel plot.

Supplementary Figure 5 Sensitivity analysis for any margin-related reoperation after excluding large registry or administrative database studies. The upper panel shows the random-effects Mantel–Haenszel forest plot, and the lower panel shows the corresponding funnel plot.

Supplementary Figure 6 Sensitivity analysis for close margin after adding studies with broader or less directly comparable close-margin definitions. The upper panel shows the random-effects Mantel–Haenszel forest plot, and the lower panel shows the corresponding funnel plot.

Supplementary Figure 7 Sensitivity analysis for negative/clear margin after replacing the potentially overlapping Vohra dataset with the Abidi dataset. The upper panel shows the random-effects Mantel–Haenszel forest plot, and the lower panel shows the corresponding funnel plot. For this favorable outcome, an RR greater than 1 favors OBCS.

Supplementary Figure 8 Sensitivity analysis for negative/clear margin after adding studies with broader, non-standard, or less directly comparable definitions. The upper panel shows the random-effects Mantel–Haenszel forest plot, and the lower panel shows the corresponding funnel plot. For this favorable outcome, an RR greater than 1 favors OBCS.

Supplementary Figure 9 Sensitivity analysis for overall complications after excluding large registry or administrative database studies. The upper panel shows the random-effects Mantel–Haenszel forest plot, and the lower panel shows the corresponding funnel plot. BCS, breast-conserving surgery; CI, confidence interval; M-H, Mantel–Haenszel; OBCS, oncoplastic breast-conserving surgery; RR, risk ratio; SE, standard error.
