# Supplementary material for: Margin status, re-excision, and completion mastectomy after oncoplastic versus conventional breast-conserving surgery for breast cancer: a systematic review and meta-analysis
Source: Front Oncol. 2026 Sep 15;16:1934279. doi: 10.3389/fonc.2026.1934279 (PMC13619401; doi:10.3389/fonc.2026.1934279)
Supplement: Supplementary file 1 [file DataSheet1.docx]

**Supplementary Methods: Literature search strategy**

We systematically searched PubMed/MEDLINE, Embase, Web of Science Core Collection, Scopus, and the Cochrane Library to identify comparative studies evaluating margin status and margin-related reoperation after oncoplastic versus conventional breast-conserving surgery for breast cancer. The searches were performed from database inception to 25 June 2026. No publication-year restriction was applied, and no language restriction was applied at the database-search stage. In addition, the reference lists of all included studies and relevant reviews were manually screened to identify further eligible studies.

Because eligible studies may have described oncoplastic breast-conserving surgery using different terms, such as “oncoplastic breast surgery,” “oncoplastic breast-conserving surgery,” “therapeutic mammoplasty,” “volume displacement,” “volume replacement,” or “oncoplastic reduction,” the search strategy was designed to balance sensitivity and specificity. Final eligibility was determined according to the prespecified PICO framework and inclusion/exclusion criteria.

**1. PubMed**

**Database: PubMed/MEDLINE**

Date range: from database inception to 25 June 2026

Search field: Title/Abstract and related indexing terms

**Search strategy:**

(("breast cancer" OR "breast neoplasm" OR "breast neoplasms" OR "breast carcinoma" OR "breast tumor" OR "breast tumors" OR "breast tumour" OR "breast tumours" OR "mammary carcinoma" OR "ductal carcinoma in situ" OR DCIS)
AND
(oncoplastic OR oncoplast* OR OBCS OR OPBS OR "oncoplastic breast surgery" OR "oncoplastic breast-conserving surgery" OR "oncoplastic breast conserving surgery" OR "oncoplastic surgery" OR "oncoplastic technique" OR "oncoplastic techniques" OR "oncoplastic reduction" OR "therapeutic mammoplasty" OR "therapeutic mammaplasty" OR "therapeutic reduction mammoplasty" OR "therapeutic reduction mammaplasty" OR "volume displacement" OR "volume replacement" OR "partial breast reconstruction" OR "glandular flap" OR "local flap" OR "breast reshaping")
AND
("breast-conserving surgery" OR "breast conserving surgery" OR "breast conservation surgery" OR "breast-conservation surgery" OR "breast-conserving therapy" OR "breast conserving therapy" OR BCS OR lumpectomy OR "wide local excision" OR WLE OR "partial mastectomy" OR segmentectomy OR quadrantectomy)
AND
("surgical margin" OR "surgical margins" OR "resection margin" OR "resection margins" OR "positive margin" OR "positive margins" OR "close margin" OR "close margins" OR "negative margin" OR "negative margins" OR "clear margin" OR "clear margins" OR "tumor-free margin" OR "tumor free margin" OR "tumour-free margin" OR "tumour free margin" OR "involved margin" OR "involved margins" OR "margin status" OR "margin involvement" OR "margin positivity" OR "re-excision" OR reexcision OR "re-excisions" OR "repeat excision" OR "repeat surgery" OR reoperation OR reoperations OR "re-operation" OR "re-operations" OR "second operation" OR "secondary surgery" OR "additional surgery" OR "completion mastectomy" OR "conversion to mastectomy" OR "secondary mastectomy" OR "salvage mastectomy"))
NOT
("systematic review" OR "meta-analysis" OR "meta analysis" OR editorial OR letter OR comment OR "case report" OR "case reports" OR protocol)

**2. Embase**

**Database: Embase**

Date range: from database inception to 25 June 2026

**Search strategy:**

(("breast cancer" OR "breast neoplasm" OR "breast neoplasms" OR "breast carcinoma" OR "breast tumor" OR "breast tumors" OR "breast tumour" OR "breast tumours" OR "mammary carcinoma" OR "ductal carcinoma in situ" OR DCIS)
AND
(oncoplastic OR oncoplast* OR OBCS OR OPBS OR "oncoplastic breast surgery" OR "oncoplastic breast-conserving surgery" OR "oncoplastic breast conserving surgery" OR "oncoplastic surgery" OR "oncoplastic technique" OR "oncoplastic techniques" OR "oncoplastic reduction" OR "therapeutic mammoplasty" OR "therapeutic mammaplasty" OR "therapeutic reduction mammoplasty" OR "therapeutic reduction mammaplasty" OR "volume displacement" OR "volume replacement" OR "partial breast reconstruction" OR "glandular flap" OR "local flap" OR "breast reshaping")
AND
("breast-conserving surgery" OR "breast conserving surgery" OR "breast conservation surgery" OR "breast-conservation surgery" OR "breast-conserving therapy" OR "breast conserving therapy" OR BCS OR lumpectomy OR "wide local excision" OR WLE OR "partial mastectomy" OR segmentectomy OR quadrantectomy)
AND
("surgical margin" OR "surgical margins" OR "resection margin" OR "resection margins" OR "positive margin" OR "positive margins" OR "close margin" OR "close margins" OR "negative margin" OR "negative margins" OR "clear margin" OR "clear margins" OR "tumor-free margin" OR "tumor free margin" OR "tumour-free margin" OR "tumour free margin" OR "involved margin" OR "involved margins" OR "margin status" OR "margin involvement" OR "margin positivity" OR "re-excision" OR reexcision OR "re-excisions" OR "repeat excision" OR "repeat surgery" OR reoperation OR reoperations OR "re-operation" OR "re-operations" OR "second operation" OR "secondary surgery" OR "additional surgery" OR "completion mastectomy" OR "conversion to mastectomy" OR "secondary mastectomy" OR "salvage mastectomy"))
NOT
("systematic review" OR "meta analysis" OR "meta-analysis" OR editorial OR letter OR comment OR "case report" OR "case reports" OR protocol)

**3. Web of Science Core Collection**

**Database: Web of Science Core Collection**

Date range: from database inception to 25 June 2026

Search field: Topic (TS)

**Search strategy:**

TS=(("breast cancer" OR "breast neoplasm" OR "breast neoplasms" OR "breast carcinoma" OR "breast tumor" OR "breast tumors" OR "breast tumour" OR "breast tumours" OR "mammary carcinoma" OR "ductal carcinoma in situ" OR DCIS)
AND
(oncoplastic OR oncoplast* OR OBCS OR OPBS OR "oncoplastic breast surgery" OR "oncoplastic breast-conserving surgery" OR "oncoplastic breast conserving surgery" OR "oncoplastic surgery" OR "oncoplastic technique" OR "oncoplastic techniques" OR "oncoplastic reduction" OR "therapeutic mammoplasty" OR "therapeutic mammaplasty" OR "therapeutic reduction mammoplasty" OR "therapeutic reduction mammaplasty" OR "volume displacement" OR "volume replacement" OR "partial breast reconstruction" OR "glandular flap" OR "local flap" OR "breast reshaping")
AND
("breast-conserving surgery" OR "breast conserving surgery" OR "breast conservation surgery" OR "breast-conservation surgery" OR "breast-conserving therapy" OR "breast conserving therapy" OR BCS OR lumpectomy OR "wide local excision" OR WLE OR "partial mastectomy" OR segmentectomy OR quadrantectomy)
AND
("surgical margin" OR "surgical margins" OR "resection margin" OR "resection margins" OR "positive margin" OR "positive margins" OR "close margin" OR "close margins" OR "negative margin" OR "negative margins" OR "clear margin" OR "clear margins" OR "tumor-free margin" OR "tumor free margin" OR "tumour-free margin" OR "tumour free margin" OR "involved margin" OR "involved margins" OR "margin status" OR "margin involvement" OR "margin positivity" OR "re-excision" OR reexcision OR "re-excisions" OR "repeat excision" OR "repeat surgery" OR reoperation OR reoperations OR "re-operation" OR "re-operations" OR "second operation" OR "secondary surgery" OR "additional surgery" OR "completion mastectomy" OR "conversion to mastectomy" OR "secondary mastectomy" OR "salvage mastectomy"))
NOT
TS=("systematic review" OR "meta-analysis" OR "meta analysis" OR editorial OR letter OR comment OR "case report" OR "case reports" OR protocol)

**4. Scopus**

**Database: Scopus**

Date range: from database inception to 25 June 2026

Search field: TITLE-ABS-KEY

**Search strategy:**

TITLE-ABS-KEY(("breast cancer" OR "breast neoplasm" OR "breast neoplasms" OR "breast carcinoma" OR "breast tumor" OR "breast tumors" OR "breast tumour" OR "breast tumours" OR "mammary carcinoma" OR "ductal carcinoma in situ" OR DCIS)
AND
(oncoplastic OR oncoplast* OR OBCS OR OPBS OR "oncoplastic breast surgery" OR "oncoplastic breast-conserving surgery" OR "oncoplastic breast conserving surgery" OR "oncoplastic surgery" OR "oncoplastic technique" OR "oncoplastic techniques" OR "oncoplastic reduction" OR "therapeutic mammoplasty" OR "therapeutic mammaplasty" OR "therapeutic reduction mammoplasty" OR "therapeutic reduction mammaplasty" OR "volume displacement" OR "volume replacement" OR "partial breast reconstruction" OR "glandular flap" OR "local flap" OR "breast reshaping")
AND
("breast-conserving surgery" OR "breast conserving surgery" OR "breast conservation surgery" OR "breast-conservation surgery" OR "breast-conserving therapy" OR "breast conserving therapy" OR BCS OR lumpectomy OR "wide local excision" OR WLE OR "partial mastectomy" OR segmentectomy OR quadrantectomy)
AND
("surgical margin" OR "surgical margins" OR "resection margin" OR "resection margins" OR "positive margin" OR "positive margins" OR "close margin" OR "close margins" OR "negative margin" OR "negative margins" OR "clear margin" OR "clear margins" OR "tumor-free margin" OR "tumor free margin" OR "tumour-free margin" OR "tumour free margin" OR "involved margin" OR "involved margins" OR "margin status" OR "margin involvement" OR "margin positivity" OR "re-excision" OR reexcision OR "re-excisions" OR "repeat excision" OR "repeat surgery" OR reoperation OR reoperations OR "re-operation" OR "re-operations" OR "second operation" OR "secondary surgery" OR "additional surgery" OR "completion mastectomy" OR "conversion to mastectomy" OR "secondary mastectomy" OR "salvage mastectomy"))
AND NOT
TITLE-ABS-KEY("systematic review" OR "meta-analysis" OR "meta analysis" OR editorial OR letter OR comment OR "case report" OR "case reports" OR protocol)

**5. Cochrane Library**

**Database: Cochrane Library**

Date range: from database inception to 25 June 2026

**Search strategy:**

(("breast cancer" OR "breast neoplasm" OR "breast neoplasms" OR "breast carcinoma" OR "breast tumor" OR "breast tumors" OR "breast tumour" OR "breast tumours" OR "mammary carcinoma" OR "ductal carcinoma in situ" OR DCIS)
AND
(oncoplastic OR oncoplast* OR OBCS OR OPBS OR "oncoplastic breast surgery" OR "oncoplastic breast-conserving surgery" OR "oncoplastic breast conserving surgery" OR "oncoplastic surgery" OR "oncoplastic technique" OR "oncoplastic techniques" OR "oncoplastic reduction" OR "therapeutic mammoplasty" OR "therapeutic mammaplasty" OR "therapeutic reduction mammoplasty" OR "therapeutic reduction mammaplasty" OR "volume displacement" OR "volume replacement" OR "partial breast reconstruction" OR "glandular flap" OR "local flap" OR "breast reshaping")
AND
("breast-conserving surgery" OR "breast conserving surgery" OR "breast conservation surgery" OR "breast-conservation surgery" OR "breast-conserving therapy" OR "breast conserving therapy" OR BCS OR lumpectomy OR "wide local excision" OR WLE OR "partial mastectomy" OR segmentectomy OR quadrantectomy)
AND
("surgical margin" OR "surgical margins" OR "resection margin" OR "resection margins" OR "positive margin" OR "positive margins" OR "close margin" OR "close margins" OR "negative margin" OR "negative margins" OR "clear margin" OR "clear margins" OR "tumor-free margin" OR "tumor free margin" OR "tumour-free margin" OR "tumour free margin" OR "involved margin" OR "involved margins" OR "margin status" OR "margin involvement" OR "margin positivity" OR "re-excision" OR reexcision OR "re-excisions" OR "repeat excision" OR "repeat surgery" OR reoperation OR reoperations OR "re-operation" OR "re-operations" OR "second operation" OR "secondary surgery" OR "additional surgery" OR "completion mastectomy" OR "conversion to mastectomy" OR "secondary mastectomy" OR "salvage mastectomy"))
NOT
("systematic review" OR "meta-analysis" OR "meta analysis" OR editorial OR letter OR comment OR "case report" OR "case reports" OR protocol)
