# Supplementary material for: Margin status, re-excision, and completion mastectomy after oncoplastic versus conventional breast-conserving surgery for breast cancer: a systematic review and meta-analysis
Source: Front Oncol. 2026 Sep 15;16:1934279. doi: 10.3389/fonc.2026.1934279 (PMC13619401; doi:10.3389/fonc.2026.1934279)
Supplement: Supplementary file 2 [file Table1.docx]

| Supplementary Table S1. Detailed baseline and surgical characteristics of included studies | | | | | | | | | | | | | | | | | | | |
| --- | --- | --- | --- | --- | --- | --- | --- | --- | --- | --- | --- | --- | --- | --- | --- | --- | --- | --- | --- |
| Study | Country/ Region | Study design | Disease / stage | Total n | OBCS n | C-BCS n | Age, OBCS M (IQR/range) or mean ± SD | Age, C-BCS M (IQR/range) or mean ± SD | Tumor size, OBCS M (IQR/range) or mean ± SD | Tumor size, C-BCS M (IQR/range) or mean ± SD | DCIS, OBCS n/N (%) | DCIS, C-BCS n/N (%) | ILC, OBCS n/N (%) | ILC, C-BCS n/N (%) | NACT, OBCS n/N (%) | NACT, C-BCS n/N (%) | RT, OBCS n/N (%) | RT, C-BCS n/N (%) | FU (mo) |
| Abdelkader et al. [17] | Egypt | Single-center, retrospective comparative study | Primary invasive BC/DCIS | 310 | 155 | 155 | NR | NR | 3.20 ± 0.88 cm | 2.12 ± 0.55 cm | 0/155 (0) | 6/155 (3.9) | 18/155 (11.6) | 0/155 (0) | 43/155 (27.7) | 41/155 (26.5) | NR | NR | NR |
| Abidi et al. [18] | Pakistan | Retrospective cohort study | Early/LABC breast cancer | 422† | 249 | 173 | 49.9 | 51.1 | 2.26 ± 1.66 cm | 1.94 ± 1.28 cm | 23/249 (10.0) | 22/173 (15.9) | 2/249 (0.9) | 2/173 (1.4) | 53/249 (21.3) | 45/173 (26.0) | NR | NR | NR |
| Almeida et al. [19] | Brazil | Single-center, retrospective cohort study | Breast cancer treated with BCS | 866 | 98 | 768 | NR | NR | NR | NR | 13/98 (13.3) | 60/768 (7.8) | 5/98 (5.1) | 22/768 (2.9) | 22/98 (22.4) | 75/768 (9.8) | NR | NR | 50.4 |
| Almojel et al. [20] | Saudi Arabia | Single-center, retrospective cohort study | Breast cancer, stages 0–IV | 237 | 137 | 100 | 49.2 ± 10.4 | 56.4 ± 12.3 | 34.2 ± 22.2 mm | 38.3 ± 23.0 mm | 16/137 (11.7) | 4/100 (4.0) | 13/137 (9.5) | 11/100 (11.0) | 74/137 (54.0) | 54/100 (54.0) | 93/137 (67.9) | 65/100 (65.0) | 20.4 (8.4–36.0) |
| André et al. [21] | Sweden | Multicenter, register-based retrospective cohort study | Primary invasive BC; T1–T3; BCS+RT | 4178 | 458 | 3720 | 59 (29–85) / 58 (30–81) | 63 (23–91) | 18 (7–100) / 21 (2–86) mm | 16 (1–80) mm | 0/458 (0) | 0/3720 (0) | 48/458 (10.5) | 399/3720 (10.7) | 73/458 (15.9) | 168/3720 (4.5) | 458/458 (100) | 3720/3720 (100) | 64 (24–110) |
| Atallah et al.[22] | Lebanon | Single-center, retrospective case-control study | Invasive/in situ breast cancer; primary surgery | 277 | 193 | 84 | 53.56 ± 12.52 | 52.07 ± 11.47 | 1.89 ± 1.11 cm | 1.86 ± 0.95 cm | 24/184 (13.0) | 23/61 (37.7) | 53/159 (33.3) | 23/61 (37.7) | 0/193 (0) | 0/84 (0) | 193/193 (100) | 84/84 (100) | 66 (24–108) / 72 (24–120) |
| Azhar et al. [23] | Indonesia | Multicenter, retrospective cohort study | Stage I–III breast cancer | 82 | 39 | 43 | 39.25 ± 4.90 | 39.86 ± 5.11 | 34.72 ± 7.00 cm³ | 22.35 ± 7.80 cm³ | 9/39 (23.1) | 10/43 (23.3) | 5/39 (12.8) | 2/43 (4.7) | 24/39 (61.5) | 30/43 (69.8) | NR | NR | 30 d |
| Bali et al. [24] | United Kingdom | Single-center, retrospective cohort study | Invasive carcinoma/DCIS treated with BCS | 201 | 35 | 166 | 57.2 ± 11.4 | 60.9 ± 12.7 | 30.4 ± 17.5 mm | 19.0 ± 11.5 mm | 27/35 (77.1)‡ | 105/166 (63.3)‡ | 3/35 (8.6) | 18/166 (10.8) | 9/35 (25.7) | 12/166 (7.2) | 30/35 (85.7) | 150/166 (90.4) | NR |
| Bargon et al. [13] | Netherlands | Prospective multicenter longitudinal cohort study | Invasive breast cancer/DCIS treated with BCS | 1918 | 290 | 1628 | 57.1 (31–85) | 59.9 (24–91) | 18.1 ± 11.6 mm | 14.3 ± 8.6 mm | 39/290 (13.4) | 219/1628 (13.5) | NR | NR | 42/290 (14.5) | 136/1628 (8.4) | 287/290 (99.0) | 1603/1628 (98.5) | 24 |
| Behluli et al. [25] | Germany/Switzerland | Multicenter, retrospective comparative study | Primary nonmetastatic invasive/in situ/mixed BC | 343 | 52 | 291 | NR | NR | NR | NR | NR | NR | NR | NR | 0/52 (0) | 0/291 (0) | NR | NR | NR |
| Caixeiro et al. [26] | Portugal | Single-center, retrospective cohort study | Breast cancer; pTis/pT0/pT1/pT2 | 215 | 90 | 125 | 57 (15; 36–74) | 60 (18; 33–84) | 15 (8; 5–30) mm | 11 (7.3; 1–30) mm | 7/90 (7.8) | 24/125 (19.2) | 2/90 (2.2) | 7/125 (5.6) | 10/90 (11.1) | 7/125 (5.6) | NR | NR | 89.8 (3–158) |
| Calì Cassi et al. [27] | Italy | Single-center, retrospective observational cohort study | Breast cancer treated with lumpectomy | 215 procedures† | 61 | 154 | 54.7 ± 12.6 | 63.2 ± 12.1 | 1.3 ± 0.7 cm | 1.4 ± 0.9 cm | NR | NR | NR | NR | NR | NR | 45/61 (73.8) | 117/154 (76.0) | 44.8 ± 16.0 / 43.3 ± 21.1 |
| Caziuc et al. [28] | Romania | Single-center, retrospective comparative study | DCIS or invasive BC with DCIS component | 76 | 29 | 47 | 45.12 ± 2.19 | 51.35 ± 2.52 | 3.19 ± 0.76 cm | 1.20 ± 0.89 cm | 29/29 (100)‡ | 47/47 (100)‡ | NR | NR | 0/29 (0) | 0/47 (0) | 29/29 (100) | 47/47 (100) | NR |
| Çelik et al. [29] | Türkiye | Single-center, retrospective cohort study | Breast cancer with DCIS component | 163 | 40 | 123 | 50 (29–65) | 52 (25–68) | 16 (3–45) mm | 15 (4–50) mm | 40/40 (100)‡ | 123/123 (100)‡ | NR | NR | 11/40 (27.5) | 33/123 (26.8) | 40/40 (100) | 123/123 (100) | 24 (18–36) |
| Chakravorty et al. [30] | United Kingdom | Single-center, retrospective comparative cohort study | Breast cancer treated with BCS | 590 | 150 | 440 | 59 (26–83) | 61 (27–90) | 21 (1–98) mm | 18 (2–98) mm | 36/150 (24.0)‡ | 20/440 (4.5)‡ | NR | NR | 38/150 (25.0) | 14/440 (3.0) | 135/150 (90.0) | 407/440 (92.5) | 28 (6–81) |
| Chauhan et al. [31] | India | Single-center, prospective comparative study with historical controls | Early breast cancer; T1/T2, N0/N1 | 79 | 33 | 46 | 39.4 ± 6.7 | 42.1 ± 7.2 | 43.2 (16–76) cm³† | 36.4 (15–62) cm³† | NR | NR | NR | NR | 0/33 (0) | 0/46 (0) | 33/33 (100) | 46/46 (100) | 18 (6–30) / 38 (12–64) |
| Chauhan et al. [32] | India | Single-center, prospective comparative study with historical controls | LABC after NACT; cT3/T4, N0–N2 | 100 | 57 | 43 | 46.9 ± 13.1 | 54.3 ± 5.3 | 4.4 ± 1.6 cm (post-NACT) | 2.3 ± 1.1 cm (post-NACT) | NR | NR | 1/57 (1.8) | 1/43 (2.3) | 57/57 (100) | 43/43 (100) | 57/57 (100) | 43/43 (100) | 18 (6–30) / 34 (14–44) |
| Crown et al. [33] | United States | Single-center, retrospective before-after cohort study | Invasive or noninvasive breast carcinoma | 610† | 329 | 281 | NR | NR | 15.4 mm† | 12.7 mm† | NR | NR | NR | NR | NR | NR | NR | NR | NR |
| Çulcu et al. [34] | Türkiye | Single-center, retrospective cohort study | Invasive breast cancer; pure DCIS and NACT excluded | 460 | 237 | 223 | 51.8 ± 10.08 | 50.99 ± 11.12 | 24.35 ± 9.96 mm | 25.68 ± 11.65 mm | 0/237 (0) | 0/223 (0) | NR | NR | 0/237 (0) | 0/223 (0) | 237/237 (100) | 223/223 (100) | 52 |
| De Lorenzi et al. [35] | Italy | Single-center, retrospective cohort study | Pure DCIS treated with conservation + RT | 419 | 44 | 375 | NR | NR | NR | NR | 44/44 (100) | 375/375 (100) | 0/44 (0) | 0/375 (0) | 0/44 (0) | 0/375 (0) | 44/44 (100) | 375/375 (100) | 92.4 / 110.4 |
| Di Micco et al. [36] | United Kingdom | Single-center, retrospective cohort study | Large-breasted women with early (<3 cm) unilateral BC | 157 | 70 | 87 | 55 (33–77) | 63.9 (43.9–83.8) | 28 (0–136) mm | 20 (0–80) mm | 4/70 (5.7) | 10/87 (11.5) | 6/70 (8.6) | 8/87 (9.2) | 14/70 (20.0) | 8/87 (9.2) | 70/70 (100) | 87/87 (100) | 36 (9.8–76) |
| Down et al. [37] | United Kingdom | Single-center, retrospective cohort study | Early invasive BC or DCIS requiring BCS | 158 procedures† | 37 | 121† | 57 (35–86) | 61 (34–84) | 23.9 mm | 17.6 mm | 4/37 (10.8) | 27/121 (22.3) | 0/37 (0) | 8/121 (6.6) | NR | NR | 33/37 (89.2) | 104/121 (85.9) | 23.7 overall; 29.3 / 22.1 |
| Falade et al. [38] | United States | Single-center, retrospective cohort study | Stage I–III invasive lobular carcinoma | 494 | 168 | 326 | 60.5 / 58.1† | 62.5 | 2.2 / 3.3 cm† | 2.2 cm | 0/168 (0) | 0/326 (0) | 168/168 (100) | 326/326 (100) | NR | NR | NR | NR | 8.0 ± 6.5 y overall |
| Garreffa et al. [39] | United Kingdom | Single-center, retrospective cohort study | Invasive or in situ BC treated with BCS | 123 breasts† | 63 breasts† | 60 | 55.82 (29–80) | 58.98 (29–80) | 30.14 mm | 19.7 mm | 8/63 (12.7) | 6/60 (10.0) | 8/63 (12.7) | 4/60 (6.7) | 13/63 (20.6) | 8/60 (13.3) | 63/63 (100) | 51/60 (85.0) | NR |
| Giacalone et al. [40] | France | Prospective comparative study | Breast cancer, tumor diameter ≥15 mm; eligible for either approach | 74 | 31 | 43 | 51.3 ± 12.1 | 58.5 ± 1.7† | 20 (15–60) mm radiologic | 20 (15–60) mm radiologic | 8/31 (25.8) | 6/43 (14.0) | 1/31 (3.2) | 3/43 (7.0) | NR | NR | NR | NR | NR |
| Gulcelik et al. [41] | Türkiye | Single-center, prospective comparative study | Invasive BC; stages I–IIIA | 268 | 106 | 162 | 53 ± 12.4 | 51 ± 10.8 | 2.7 cm | 2.4 cm | 0/106 (0) | 0/162 (0) | NR | NR | NR | NR | 106/106 (100) | 162/162 (100) | 33 (16–88) / 37 (20–124) |
| Heeg et al. [14] | Denmark | Nationwide population-based cohort study with PSM | Invasive BC without distant metastasis | 18188 | 5003 | 13185 | 59.9 ± 11.5 | 62.1 ± 11.5 | NR | NR | NR | NR | 549/5003 (11.0) | 1339/13185 (10.2) | 0/5003 (0)† | 0/13185 (0)† | NR | NR | 2 mo† |
| Kaur et al. [42] | Italy | Prospective nonrandomized comparative study | Breast cancer; pTis/T1/T2 | 60 | 30 | 30 | 48.73 (30–75) | 55.76 (34–81) | NR | NR | 4/30 (13.3) | 11/30 (36.7) | NR | NR | NR | NR | NR | NR | NR |
| Kelemen et al. [43] | Hungary | Single-center, retrospective cohort study | Stage 0–III breast cancer | 700 | 350 | 350 | 58 (31–85) | 59 (29–86) | NR | NR | 22/350 (6.3) | 30/350 (8.6) | 35/350 (10.0) | 21/350 (6.0) | 27/350 (7.7) | 1/350 (0.3) | 350/350 (100) | 350/350 (100) | 51 / 52 |
| Kilmer et al. [44] | United States | National claims database retrospective cohort study | Breast cancer; matched lumpectomy vs OBRS cohort | 30268 | 15134 | 15134 | Matched age ranges; mean NR | Matched age ranges; mean NR | NR | NR | NR | NR | NR | NR | 1421/15134 (9.4) | 1421/15134 (9.4) | NR | NR | 12 mo† |
| Lima et al. [45] | Brazil | Multicenter, retrospective real-world cohort study | Stage 0–III breast cancer; BCS + adjuvant RT | 685 | 135 | 550 | 53.3 ± 10.6 | 59.5 ± 13.3 | NR | NR | 10/135 (7.4) | 66/550 (12.0) | 19/135 (14.1) | 47/550 (8.5) | 42/135 (31.1) | 100/550 (18.2) | 130/135 (96.3) | 504/550 (91.6) | 42 |
| Lim et al. [46] | Singapore | Matched case-control study | Histologically proven breast cancer | 22 | 11 | 11 | 49.9 ± 11.7 | 46.9 ± 9.50 | 28.9 ± 17.7 mm | 29.5 ± 8.48 mm | 2/11 (18.2) | 2/11 (18.2) | 0/11 (0) | 0/11 (0) | 1/11 (9.1) | 0/11 (0) | 10/11 (90.9) | 10/11 (90.9) | 24 (11–29) / 50 (10–60) |
| Lopez Rios et al. [47] | Canada | Single-center, retrospective cohort study | Breast cancer; stage IV/metastatic/recurrent excluded | 1290 | 307 | 983 | 56.45 ± 11.69 | 61.11 ± 11.98 | 2.58 ± 2.45 cm | 1.86 ± 1.11 cm | 26/307 (8.5) | 168/983 (17.1) | 41/307 (13.4) | 92/983 (9.4) | 30/307 (9.8) | 29/983 (3.0) | 283/307 (92.2) | 871/983 (88.6) | 55.7 / 66.5 |
| Mukhtar et al. [48] | United States | Single-center, retrospective cohort study | Stage I–III invasive lobular carcinoma; attempted BCS | 365 tumors† | 88 | 277 | NR | NR | 2.5 / 3.0 cm† | 2.2 cm | 0/88 (0) | 0/277 (0) | 88/88 (100) | 277/277 (100) | NR | NR | NR | NR | NR |
| Nanda et al. [49] | United Kingdom | Single-center, retrospective cohort study | Breast cancer treated with BCS | 159 | 79 | 80 | 51.63 ± 8.83 | 58.18 ± 10.87 | 31.04 ± 20.56 mm | 24.44 ± 16.83 mm | 5/79 (6.3) | 4/80 (5.0) | 13/79 (16.5) | 9/80 (11.3) | 15/79 (19.0) | 5/80 (6.3) | 74/79 (93.7) | 66/80 (82.5) | 89.5 / 87 |
| Niinikoski et al. [50] | Finland | Single-center, retrospective cohort study | Primary invasive BC/DCIS; BCS | 1800 | 611 | 1189 | NR | NR | NR | NR | 29/611 (4.7) | 64/1189 (5.4) | 76/611 (12.4) | 136/1189 (11.4) | 0/611 (0) | 0/1189 (0) | 580/611 (94.9) | 1126/1189 (94.7) | 75 (2–94) |
| Nisiri et al. [51] | Iran | Single-center, comparative retrospective study | Stage I–III unilateral breast cancer | 317 | 154 | 163 | NR | NR | T1/T2/T3: 48/98/8 | T1/T2/T3: 57/101/5 | NR | NR | NR | NR | NR | NR | NR | NR | 24 |
| Oh et al. [52] | South Korea | Single-center, retrospective cohort study with PSM | Early breast cancer; BCS+RT | 742 | 371 | 371 | 48.9 ± 9.2 | 49.4 ± 9.7 | 3.0 ± 1.7 cm | 2.3 ± 1.3 cm | NR | NR | NR | NR | 94/371 (25.3) | 86/371 (23.2) | 371/371 (100) | 371/371 (100) | 84.4 ± 22.7 / 87.9 ± 24.8 |
| Rose et al. [53] | Denmark | Population-based retrospective cohort study | Primary invasive breast cancer; BCS | 1596 | 197 | 1399 | 57 (31–80) | 61 (24–93) | NR | NR | 0/197 (0) | 0/1399 (0) | NR | NR | NR | NR | NR | NR | 49.2 / 67.2† |
| Sagiroglu et al. [54] | Türkiye | Single-center, retrospective comparative study | Unilateral early BC; stage I–IIB | 288 | 138 | 150 | NR | NR | 2.12 ± 1.31 cm | 1.99 ± 0.79 cm | 60/138 (43.5)‡ | 80/150 (53.3)‡ | 4/138 (2.9) | 12/150 (8.0) | 0/138 (0) | 0/150 (0) | 138/138 (100) | 150/150 (100) | 74 (7–89) |
| Saha et al. [55] | India | Randomized controlled trial | Early breast cancer; tumor ≤4 cm | 104 | 52 | 52 | 47.52 ± 10.41 / 48.96 ± 10.50 | 49.88 ± 11.13 / 49.46 ± 14.05 | 2.7 (1–4) / 2.5 (1.1–3.8) cm | 2.55 (1.3–4) / 2.3 (0–4) cm | 0/52 (0)† | 3/52 (5.8)† | NR | NR | 4/52 (7.7) | 1/52 (1.9) | NR | NR | 54 (12–70) |
| Shah et al. [56] | United States | National claims database retrospective cohort study | Adult female breast cancer; BCT + RT | 53165 | 1552 | 51613 | NR; age categories | NR; age categories | NR | NR | NR | NR | NR | NR | NR | NR | 1552/1552 (100) | 51613/51613 (100) | ≥6 mo |
| Tenofsky et al. [57] | United States | Single-surgeon, retrospective cohort study | Female breast cancer treated with lumpectomy | 142 | 58 | 84 | 60.9 ± 11.8 | 65.2 ± 12.7 | 11.0 ± 9.8 mm | 10.8 ± 7.3 mm | NR | NR | NR | NR | NR | NR | 54/58 (93.1) | 59/84 (70.2) | 24.6 ± 10.2 / 26.2 ± 16.5 |
| Tokui et al. [58] | Japan | Single-center, retrospective cohort study | Stage 0–III breast cancer; no neoadjuvant therapy | 1185 | 145 | 1040 | ≤40 y: 30/145 (20.7) | ≤40 y: 68/1040 (6.5) | pT2/3: 65/145 (44.8) | pT2/3: 326/1040 (31.3) | 18/145 (12.4)† | 114/1040 (11.0)† | NR | NR | 0/145 (0) | 0/1040 (0) | NR | NR | 61 (1–104) |
| Vieira et al. [59] | Brazil | Matched case-control study | LABC after NACT; BCS | 78 | 26 | 52 | 49.75 ± 1.80 | 48.27 ± 1.69 | 5.26 ± 1.66 cm | 5.25 ± 1.52 cm | NR | NR | NR | NR | 26/26 (100) | 52/52 (100) | 26/26 (100) | 50/52 (96.2) | 60.01 ± 18.19 / 64.88 ± 24.53 |
| Vohra et al. [60] | Pakistan | Multicenter, retrospective cohort study | Stage I–III breast cancer | 481 | 277 | 204 | 49 | 51 | 148.8 cm³ | 90.4 cm³ | 13/277 (4.7) | 6/204 (2.9) | 6/277 (2.2) | 0/204 (0) | 108/277 (39.0) | 58/204 (28.4) | NR | NR | 1 (30 d) |
| Wijesinghe et al. [61] | Sri Lanka | Two-center, retrospective cohort study | Primary non-metastatic breast cancer; early/LABC | 127 | 73 | 54 | 51 (39–68) | 56 (42–74) | 4.2 (1.2–5.2) cm | 2.4 (1.0–2.6) cm | NR | NR | NR | NR | NR | NR | NR | NR | 12 |
| Zhou et al. [62] | China | Single-center, retrospective comparative cohort study | Stage I–II breast cancer | 106 | 43 | 63 | 47.42 ± 9.24 | 45.19 ± 10.34 | 2.16 ± 0.83 cm | 1.91 ± 0.88 cm | 0/43 (0) | 3/63 (4.76) | NR | NR | 2/43 (4.65) | 4/63 (6.34) | NR | NR | 2 (0.25–6) |
| Note: Data are shown as n/N (%) unless otherwise indicated. Age, tumor size, study period, and follow-up are presented as reported in the original articles. † Non-standard, derived, combined-group, or incompletely reported denominator; use with caution in subgroup or sensitivity analyses. ‡ DCIS component rather than pure DCIS, or an alternative histologic category as reported in the original study. Abbreviations: BC, breast cancer; BCS, breast-conserving surgery; C-BCS, conventional breast-conserving surgery; DCIS, ductal carcinoma in situ; FU, follow-up; ILC, invasive lobular carcinoma; IQR, interquartile range; LABC, locally advanced breast cancer; NA, not applicable; NACT, neoadjuvant chemotherapy; NR, not reported; OBCS, oncoplastic breast-conserving surgery; RT, radiotherapy; SD, standard deviation. | | | | | | | | | | | | | | | | | | | |
