# Supplementary material for: Margin status, re-excision, and completion mastectomy after oncoplastic versus conventional breast-conserving surgery for breast cancer: a systematic review and meta-analysis
Source: Front Oncol. 2026 Sep 15;16:1934279. doi: 10.3389/fonc.2026.1934279 (PMC13619401; doi:10.3389/fonc.2026.1934279)
Supplement: Supplementary file 3 [file Table2.docx]

| Supplementary Table S2. Detailed margin-related, oncologic, safety, and patient-reported outcomes | | | | | | | | | | | | | | | | | | | | | | | | | | | |
| --- | --- | --- | --- | --- | --- | --- | --- | --- | --- | --- | --- | --- | --- | --- | --- | --- | --- | --- | --- | --- | --- | --- | --- | --- | --- | --- | --- |
| Study | Margin definition | Initial PM, OBCS n/N (%) | Initial PM, C-BCS n/N (%) | Final PM, OBCS n/N (%) | Final PM, C-BCS n/N (%) | Close margin, OBCS n/N (%) | Close margin, C-BCS n/N (%) | Negative margin, OBCS n/N (%) | Negative margin, C-BCS n/N (%) | Margin width, OBCS M (IQR/range) or mean ± SD | Margin width, C-BCS M (IQR/range) or mean ± SD | RE, OBCS n/N (%) | RE, C-BCS n/N (%) | CM, OBCS n/N (%) | CM, C-BCS n/N (%) | Any MRR, OBCS n/N (%) | Any MRR, C-BCS n/N (%) | LR, OBCS n/N (%) | LR, C-BCS n/N (%) | LRR, OBCS n/N (%) | LRR, C-BCS n/N (%) | Complications, OBCS n/N (%) | Complications, C-BCS n/N (%) | Complication type / severity classification | Time to RT, OBCS (d) | Time to RT, C-BCS (d) | Cosmesis / PROM |
| Abdelkader et al. [17] | FS-confirmed free margins; threshold NR | NR | NR | NR | NR | NR | NR | NR | NR | 1.24 ± 0.67 cm | 0.73 ± 0.29 cm | 12/155 (7.7)† | 30/155 (19.4)† | 0/155 (0) | NR | 12/155 (7.7)† | 30/155 (19.4)† | 5/155 (3.2) | 7/155 (4.5) | NR | NR | 34/155 (21.9)‡ | 32/155 (20.6)‡ | Types: seroma; hematoma; wound infection; wound dehiscence; lymphedema; partial nipple necrosis. Severity: formal grading NR; wound complications were managed conservatively with dressings. | NR | NR | Satisfied: 132/155 vs 90/155 |
| Abidi et al. [18] | Final histopathology; threshold NR | 5/249 (2.0)† | 31/173 (17.9)† | NR | NR | NR | NR | 244/249 (98.0)† | 142/173 (82.1)† | NR | NR | 2/249 (0.8)† | 17/173 (9.8) | 0/249 (0) | 7/173 (4.0) | 2/249 (0.8) | 17/173 (9.8) | NR | NR | NR | NR | NR | NR | NR | NR | NR | NR |
| Almeida et al. [19] | Invasive: no ink; DCIS: ≥2 mm | 6/97 (6.2) | 78/759 (10.3) | NR | NR | NR | NR | 91/97 (93.8) | 681/759 (89.7) | NR | NR | 5/98 (5.1) | 98/768 (12.7) | 4/98 (4.1) | 33/768 (4.3) | 9/98 (9.2) | 131/768 (17.1) | 6/98 (6.1) | 30/768 (3.9) | NR | NR | 35/98 (35.7) | 189/768 (24.6) | Study-defined severity: early major complications included infection and hematoma, dehiscence, or necrosis requiring reoperation; early minor complications included seroma and hematoma, dehiscence, or necrosis without reoperation. Clavien-Dindo: NR. Both index and symmetrized breasts were included; one major infection and two minor wound-dehiscence events occurred only in the symmetrized breast. | 129 | 139 | NR |
| Almojel et al. [20] | Positive margin on histopathology; threshold NR | 24/137 (17.5) | 5/100 (5.0) | NR | NR | NR | NR | 113/137 (82.5) | 95/100 (95.0) | NR | NR | 36/137 (26.3) | 13/100 (13.0) | NR | NR | 36/137 (26.3) | 13/100 (13.0) | 11/137 (8.0) | 9/100 (9.0) | 13/137 (9.5) | 9/100 (9.0) | 31/137 (22.6) | 16/100 (16.0) | Types: seroma; infection; fat necrosis; rare hematoma, delayed wound healing, and flap congestion. Severity: formal grading NR. | NR | NR | NR |
| André et al. [21] | Closest peripheral margin; PM not registered | NR | NR | NR | NR | NR | NR | NR | NR | 7 (0.1–55) / 10 (0.1–45) mm | 9 (0.1–62) mm | 27/458 (5.9) | 197/3720 (5.3) | NR | NR | 27/458 (5.9) | 197/3720 (5.3) | 4/458 (0.9) | 57/3720 (1.5) | NR | NR | NR | NR | NR | NR | NR | NR |
| Atallah et al.[22] | RE if ≤1 mm invasive or ≤2 mm DCIS | NR | NR | 0/193 (0) | 0/84 (0) | NR | NR | NR | NR | ≥1 cm: 101/169 (59.8) | ≥1 cm: 21/64 (32.8) | 9/193 (4.7) | 6/84 (7.1) | NR | NR | 9/193 (4.7) | 6/84 (7.1) | 0/193 (0) | 2/84 (2.4) | NR | NR | NR | NR | NR | NR | NR | NR |
| Azhar et al. [23] | No tumor cells on inked margin | 1/39 (2.5) | 4/43 (9.3) | NR | NR | NR | NR | 38/39 (97.4) | 39/43 (90.7) | NR | NR | 4/39 (10.3)† | 17/43 (39.5)† | NR | NR | 4/39 (10.3)† | 17/43 (39.5)† | 1/39 (2.6) | 2/43 (4.7) | NR | NR | 2/39 (5.1) | 0/43 (0) | Types: postoperative seroma; tissue maceration; fat necrosis. Severity: formal grading NR. | NR | NR | Better cosmesis in OPS; PROM NR |
| Bali et al. [24] | Unclear margins at 2, 1, 0 mm; main PM uses no tumor on ink | 2/35 (5.7) | 26/166 (15.7) | NR | NR | NR | NR | 33/35 (94.3) | 140/166 (84.3) | NR | NR | 6/35 (17.1) | 36/166 (21.7) | 0/35 (0) | 9/166 (5.4) | 6/35 (17.1) | 45/166 (27.1) | NR | NR | NR | NR | NR | NR | NR | NR | NR | NR |
| Bargon et al. [13] | Dutch guideline: free, focally positive ≤4 mm, positive >4 mm | 38/290 (13.1) | 245/1628 (15.0) | NR | NR | 31/290 (10.7)§ | 186/1628 (11.4)§ | 250/290 (86.2) | 1370/1628 (84.2) | NR | NR | 12/290 (4.1) | 79/1628 (4.9) | 2/290 (0.7) | 7/1628 (0.4) | 14/290 (4.8) | 86/1628 (5.3) | NR | NR | NR | NR | NR | NR | NR | 41 | 36 | EORTC: lower body image after OP-BCS up to 24 mo |
| Behluli et al. [25] | Margin infiltration status; threshold NR | NR | NR | NR | NR | NR | NR | NR | NR | 6.98 ± 0.59 SEM mm | 3.22 ± 0.19 SEM mm | 4/52 (7.7) | 100/291 (34.4) | 0/52 (0) | 33/291 (11.3) | 4/52 (7.7) | 100/291 (34.4) | NR | NR | NR | NR | 4/52 (7.7) | 13/291 (4.5) | Types: soft-tissue hematoma; necrosis; wound-healing disturbance. Severity: formal grading NR. | NR | NR | NR |
| Caixeiro et al. [26] | Ink on tumor; negative if >0 mm | 8/87 (9.2) | 31/121 (25.6) | NR | NR | NR | NR | 79/87 (90.8) | 90/121 (74.4) | 4.0 (8; 0–27) mm | 2.0 (3; 0–31) mm | 0/90 (0) | 4/125 (3.2) | 5/90 (5.6) | 22/125 (17.5) | 5/90 (5.6) | 26/125 (20.8) | Local/distant: 5/90 (5.6) | Local/distant: 5/125 (4.0) | NR | NR | NR | NR | NR | RT end 153.5 | RT end 131.0 | NR |
| Calì Cassi et al. [27] | Tumor margin status; re-excision threshold NR | NR | NR | NR | NR | NR | NR | NR | NR | NR | NR | 4/61 (6.5) | 20/154 (12.9) | NR | NR | 4/61 (6.5) | 20/154 (12.9) | 0/61 (0) | 1/154 (0.6) | NR | NR | 3/61 (4.9) | 2/154 (1.3) | Types: hematoma; non-healing wound. Severity: formal grading NR; no long-term complications were reported. | 5.1 mo | 4.2 mo | NR |
| Caziuc et al. [28] | DCIS PM: tumor cells within 2 mm of cut edge | 0/29 (0) | 5/47 (10.6) | NR | NR | NR | NR | 29/29 (100) | 42/47 (89.4) | 2.2 ± 0.52 | 1.9 ± 0.54 | 0/29 (0) | 5/47 (10.6) | 0/29 (0) | 2/47 (4.3) | 0/29 (0) | 5/47 (10.6) | NR | NR | NR | NR | 5/29 (17.2) | 8/47 (17.0) | Types: seroma; hematoma; wound dehiscence; infection. Severity: hematomas requiring reintervention for hemostasis were reported; formal grading NR. | NR | NR | NR |
| Çelik et al. [29] | Re-excision if high-risk DCIS <2 mm from margin | NR | NR | NR | NR | NR | NR | NR | NR | NR | NR | 3/40 (10.0) | 37/123 (30.1) | NR | NR | 3/40 (10.0) | 37/123 (30.1) | 0/40 (0) | 4/123 (3.3) | NR | NR | NR | NR | NR | NR | NR | BREAST-Q: breast 90 vs 68; psychosocial 88 vs 66; sexual 82 vs 64 |
| Chakravorty et al. [30] | MDT-based margin adequacy; threshold NR | NR | NR | NR | NR | NR | NR | NR | NR | NR | NR | 4/150 (2.7) | 59/440 (13.4) | 6/150 (4.0) | 5/440 (1.1) | 10/150 (6.7) | 64/440 (14.5) | 4/150 (2.7) | 10/440 (2.3) | NR | NR | NR | NR | NR | NR | NR | NR |
| Chauhan et al. [31] | Negative >2 mm; close <2 mm; positive at cut edge | 0/33 (0) | 5/46 (10.9) | NR | NR | 3/33 (9.1) | 6/46 (13.0) | 30/33 (90.9) | 35/46 (76.1) | 14 ± 5; closest 9 ± 3 mm | 6 ± 2; closest 3 ± 1.5 mm | 0/33 (0) | 2/46 (4.3) | 0/33 (0) | 3/46 (6.5) | 0/33 (0) | 5/46 (10.9) | NR | NR | 0/33 (0) | 6/46 (13.0) | 3/33 (9.1) | 5/46 (10.9) | Types: hematoma; surgical-site infection; partial nipple-areolar complex necrosis; infected seroma cavity; skin-flap necrosis. Severity: OBS-group events resolved with conservative measures; formal grading NR. | NR | NR | NR |
| Chauhan et al. [32] | Negative >2 mm; close <2 mm; positive at cut edge | 1/57 (1.8) | 3/43 (7.0) | NR | NR | 2/57 (3.5) | 7/43 (16.3) | 54/57 (94.7) | 33/43 (76.7) | 10.4 ± 3.1; closest 8.6 ± 2.1 mm | 6.9 ± 3.2; closest 4.9 ± 3.6 mm | 0/57 (0) | 1/43 (2.3) | 1/57 (1.8) | 2/43 (4.7) | 1/57 (1.8) | 3/43 (7.0) | NR | NR | 0/57 (0) | 5/43 (11.6) | 8/57 (14.0) | 4/43 (9.3) | Types: wound infection; partial nipple-areolar complex necrosis; skin-flap necrosis; hematoma; seroma. Severity: formal grading NR. | NR | NR | NR |
| Crown et al. [33] | Return to OR if final margin <2 mm; PM not reported | NR | NR | NR | NR | NR | NR | NR | NR | NR | NR | 60/329 (18.2)† | 90/281 (32.0)† | 6/329 (1.8)† | 5/281 (1.8)† | 60/329 (18.2)† | 90/281 (32.0)† | NR | NR | NR | NR | 17/329 (5.2)‡ | NR | Composite postoperative complication outcome as reported in the original study. Types/severity: not sufficiently detailed for standardized grading; Clavien-Dindo NR. | NR | NR | NR |
| Çulcu et al. [34] | Positive if tumor in contact with surgical margin | NR | NR | NR | NR | NR | NR | NR | NR | 11.01 ± 8.84 mm | 9.17 ± 8.80 mm | 26/237 (11.0)† | 40/223 (17.9)† | NR | NR | 26/237 (11.0)† | 40/223 (17.9)† | 6/237 (2.5) | 13/223 (5.8) | NR | NR | NR | NR | NR | NR | NR | NR |
| De Lorenzi et al. [35] | Focally involved ink on DCIS; close <1 mm; other categories 1–9 or >10 mm | 2/44 (4.5) | 13/374 (3.5) | NR | NR | 10/44 (22.7) | 67/374 (17.9) | 42/44 (95.5) | 361/374 (96.5) | NR | NR | NA† | NA† | NA† | NA† | NA† | NA† | 11/44 (25.0) | 60/375 (16.0) | 0/44 (0) | 3/375 (0.8) | NR | NR | NR | NR | NR | NR |
| Di Micco et al. [36] | Negative if >1 mm invasive and >2 mm DCIS | 3/70 (4.3)† | 14/87 (16.1)† | NR | NR | NR | NR | 67/70 (95.7)† | 73/87 (83.9)† | NR | NR | 2/70 (2.9) | 14/87 (16.1) | NR | NR | 2/70 (2.9) | 14/87 (16.1) | NR | NR | NR | NR | 24/70 (34.3) | 38/87 (43.7) | Postoperative surgical complications reported as an overall outcome. Types/severity: not consistently extractable for standardized grading; Clavien-Dindo NR. | 57 | 53 | BREAST-Q: breast satisfaction 80 vs 68; RT effects 100 vs 89 |
| Down et al. [37] | Further surgery if clear margin <5 mm | NR | NR | NR | NR | 2/37 (5.4)† | 35/121 (28.9)† | 35/37 (94.6)† | 86/121 (71.1)† | nearest 14.3 mm | nearest 6.1 mm | 1/37 (2.7) | 18/121 (14.9) | 1/37 (2.7) | 17/121 (14.0) | 2/37 (5.4) | 35/121 (28.9) | 0/37 (0) | 0/121 (0) | NR | NR | 2/37 (5.4)‡ | 3/121 (2.5)‡ | Composite postoperative complication outcome as reported. Types/severity: not sufficiently detailed for standardized grading; Clavien-Dindo NR. | NR | NR | NR |
| Falade et al. [38] | Positive margin: ink on tumor | 54/167 (32.3)† | 132/319 (41.4)† | NR | NR | NR | NR | 113/167 (67.7)† | 187/319 (58.6)† | NR | NR | 37/168 (22.0)† | 69/326 (21.2)† | 15/168 (8.9) | 85/326 (26.1) | NR | NR | NR | NR | NR | NR | NR | NR | NR | NR | NR | RFS not different by surgery type |
| Garreffa et al. [39] | Incomplete excision: ≥1 radial margin <1 mm | NR | NR | NR | NR | NR | NR | NR | NR | NR | NR | 6/63 (9.5) | 12/60 (20.0) | 2/63 (3.2) | 4/60 (6.7) | 8/63 (12.7) | 16/60 (26.7) | NR | NR | NR | NR | NR | NR | NR | NR | NR | NR |
| Giacalone et al. [40] | Negative lateral margin >2 mm; close ≤2 mm; positive at cut edge | 3/31 (9.7) | 7/43 (16.3) | NR | NR | 4/31 (12.9) | 7/43 (16.3) | 24/31 (77.4) | 29/43 (67.4) | lateral 10 (0–26); anterior 10 (0–30); posterior 5 (0–30) mm | lateral 7 (0–17); anterior 5 (0–20); posterior 5 (0–20) mm | 0/31 (0) | 1/43 (2.3) | 4/31 (12.9) | 7/43 (16.3) | 4/31 (12.9) | 8/43 (18.6) | NR | NR | NR | NR | NR | NR | NR | NR | NR | NR |
| Gulcelik et al. [41] | >2 mm and >10 mm reported; PM reported | 9/106 (8.5) | 18/162 (11.1) | NR | NR | 2/106 (1.9) | 6/162 (3.7) | 94/106 (89.0) | 134/162 (82.7) | ≥10 mm: 89/106 (84.0) | ≥10 mm: 124/162 (76.5) | 11/106 (10.4) | 16/162 (9.9)† | 0/106 (0) | 8/162 (4.9) | 11/106 (10.4) | 24/162 (14.8) | 1/106 (0.9) | 3/162 (1.9) | NR | NR | NR | NR | NR | NR | NR | NR |
| Heeg et al. [14] | Re-excision if invasive tumor on ink or DCIS <2 mm | NR | NR | NR | NR | NR | NR | NR | NR | NR | NR | 706/5003 (14.1)† | 2057/13185 (15.6)† | NR (3.2) | NR (3.7) | 706/5003 (14.1)† | 2057/13185 (15.6)† | NR | NR | NR | NR | NR | NR | NR | NR | NR | NR |
| Kaur et al. [42] | Negative >2 mm; close ≤2 mm; positive at cut edge | 1/30 (3.3) | 1/30 (3.3) | NR | NR | 4/30 (13.3) | 10/30 (33.3) | 25/30 (83.3) | 17/30 (56.7)† | 8.5 mm | 6.5 mm | NR | NR | NR | NR | NR | NR | NR | NR | NR | NR | NR | NR | NR | NR | NR | NR |
| Kelemen et al. [43] | Before 2015: ≥1 mm; after 2015: no ink invasive, ≥2 mm DCIS | NR | NR | NR | NR | NR | NR | NR | NR | 8 (0–21) mm | 4.5 (0–17) mm | 19/350 (5.4) | 38/350 (10.9) | 9/350 (2.6) | 20/350 (5.7) | 28/350 (8.0) | 58/350 (16.6) | 4/350 (1.1) | 11/350 (3.1) | 3/350 (0.9) | 2/350 (0.6) | 20/350 (5.7) | 23/350 (6.6) | Postoperative complications reported as an overall rate. Types/severity: formal grading NR. | 4.2 wk | 4.1 wk | Aesthetic 4.4 vs 3.2; body image 91.6 vs 75.0 |
| Kilmer et al. [44] | Claims-based reoperation; margin status not reported | NR | NR | NR | NR | NR | NR | NR | NR | NR | NR | 1129/15134 (7.5) | 1662/15134 (11.0) | 826/15134 (5.5) | 814/15134 (5.4) | 1840/15134 (12.2) | 2305/15134 (15.2) | NR | NR | NR | NR | 4087/15134 (27.0) | 3405/15134 (22.5) | Administrative/claims-based composite postoperative complication outcome. Severity: no Clavien-Dindo or treatment-based grading available. | NR | NR | NR |
| Lima et al. [45] | Negative: no ink on tumor; positive: inked surface | 2/113 (1.8) | 23/503 (4.6) | NR | NR | NR | NR | 111/113 (98.2) | 480/503 (95.4) | NR | NR | 2/135 (1.5)† | 22/550 (4.0)† | NR | NR | 2/135 (1.5)† | 22/550 (4.0)† | NR | NR | 3/135 (2.2) | 29/548 (5.3) | NR | NR | NR | NR | NR | NR |
| Lim et al. [46] | Shave margins obtained; threshold NR | NR | NR | NR | NR | NR | NR | NR | NR | NR | NR | 1/11 (9.1) | 2/11 (18.2) | 1/11 (9.1) | 1/11 (9.1) | NR | NR | NR | NR | 0/11 (0) | 2/11 (18.2) | 0/11 (0) | 0/11 (0) | No postoperative complications observed in either group. Severity classification: NA. | NR | NR | Excellent/good: 10/11 (90.9) vs 6/11 (54.6) |
| Lopez Rios et al. [47] | Positive margin reported; threshold NR | 32/307 (10.4) | 99/983 (10.1) | NR | NR | NR | NR | 275/307 (89.6) | 884/983 (89.9) | NR | NR | NR | NR | NR | NR | NR | NR | NR | NR | 11/307 (3.6) | 40/983 (4.1) | 130/307 (42.3)† | 227/983 (23.1)† | Postoperative surgical complications reported by type in the original study. Severity: formal Clavien-Dindo grading NR. | 3.93 ± 2.28 mo | 3.57 ± 2.14 mo | NR |
| Mukhtar et al. [48] | Positive margin: ink on tumor | 30/88 (34.1)† | 127/277 (45.8) | NR | NR | NR | NR | 58/88 (65.9)† | 150/277 (54.2) | NR | NR | 23/88 (26.1)† | 75/277 (27.1)† | 12/88 (13.6) | 81/277 (29.2) | NR | NR | NR | NR | NR | NR | NR | NR | NR | NR | NR | NR |
| Nanda et al. [49] | Adequate radial margin ≥2 mm | 7/79 (8.9)† | 21/80 (26.3)† | NR | NR | NR | NR | 72/79 (91.1) | 59/80 (73.8) | NR | NR | 5/79 (6.3) | 4/80 (5.0) | 2/79 (2.5) | 9/80 (11.3) | 7/79 (8.9) | 13/80 (16.3) | 2/78 (2.6) | 2/73 (2.7) | NR | NR | 16/79 (20.3) | 9/80 (11.3) | Types: lymphedema; hematoma; seroma; wound infection/breakdown; skin necrosis; fat necrosis; pain; glue allergy; postoperative pancreatitis. Severity: most complications were minor; 30-day return to theatre occurred only in the WLE group; formal Clavien-Dindo grading NR. | NR | NR | Symmetry surgery: 1/79 vs 8/80 |
| Niinikoski et al. [50] | 2010 transition: invasive no ink; DCIS ≥2 mm | NR | NR | NR | NR | NR | NR | NR | NR | NR | NR | 17/611 (2.8) | 42/1189 (3.5) | 39/611 (6.4) | 54/1189 (4.5) | 56/611 (9.2) | 96/1189 (8.1) | 7/471 (1.5) | 25/940 (2.7) | 3/471 (0.6) | 10/940 (1.1) | NR | NR | NR | NR | NR | NR |
| Nisiri et al. [51] | Positive: tumor/DCIS at or close to margin | 16/154 (10.4) | 30/163 (18.4) | NR | NR | 8/154 (5.2)† | 9/163 (5.5)† | 138/154 (89.6) | 133/163 (81.6) | NR | NR | 5/154 (3.2)† | 10/163 (6.1)† | 8/154 (5.2)† | 12/163 (7.4)† | 13/154 (8.4)† | 22/163 (13.5)† | NR | NR | NR | NR | NR | NR | NR | NR | NR | NR |
| Oh et al. [52] | Close/involved margin; threshold NR | NR | NR | NR | NR | 26/371 (7.0)† | 28/371 (7.5)† | 345/371 (93.0)† | 343/371 (92.5)† | NR | NR | 19/371 (5.1) | 12/371 (3.2) | NR | NR | 19/371 (5.1) | 12/371 (3.2) | NR; 5-y IBTRFS 96.9% | NR; 5-y IBTRFS 98.6% | NR | NR | 15/371 (4.0) | 14/371 (3.8) | Types: seroma; infection/abscess; hematoma; wound dehiscence. Severity: wound complications were defined by need for intervention/medication (e.g., aspiration, antibiotics, or surgical treatment); Clavien-Dindo NR. | NR | NR | NR |
| Rose et al. [53] | Radical excision ≥2 mm; no ink accepted late in study with boost | 19/190 (10.0)† | 218/1364 (16.0)† | NR | NR | NR | NR | 171/190 (90.0)† | 1146/1364 (84.0)† | NR | NR | NR | NR | NR | NR | NR | NR | NR | NR | NR | NR | NR | NR | NR | 60.6 ± 38.0 | 53.9 ± 28.0 | NR |
| Sagiroglu et al. [54] | No ink on tumor; intraoperative frozen section | 9/138 (6.5)† | 12/150 (8.0)† | NR | NR | NR | NR | NR | NR | 1.92 ± 0.98 cm | 1.66 ± 0.80 cm | 9/138 (6.5)† | 12/150 (8.0)† | NR | NR | 9/138 (6.5)† | 12/150 (8.0)† | 1/138 (0.7) | 1/150 (0.7) | NR | NR | 14 events/138† | 5 events/150† | Component postoperative complications reported as event counts rather than a patient-level overall complication outcome. Severity: formal grading NR. | NR | NR | NR |
| Saha et al. [55] | Gross 1- or 2-cm excision; re-excision for positive/close margins | NR | NR | NR | NR | NR | NR | NR | NR | NR | NR | 9/52 (17.3) | 1/52 (1.9) | NR | NR | 10/52 (19.2)† | 1/52 (1.9)† | NR | NR | NR | NR | NR; component events reported† | NR; component events reported† | Component postoperative complications reported separately; no patient-level overall complication denominator. Severity: formal grading NR. | NR | NR | No difference; composite score NR |
| Shah et al. [56] | Subsequent lumpectomy or completion mastectomy within 6 mo as surrogate for positive margins | NR | NR | NR | NR | NR | NR | NR | NR | NR | NR | NR | NR | NR | NR | 243/1552 (15.7)† | 13704/51613 (26.6)† | NR | NR | NR | NR | 507/1552 (32.7) | 6853/51613 (13.3) | Administrative/database-defined postoperative complication composite. Severity: no Clavien-Dindo or treatment-based grading available. | 84.55 ± 60.63 | 88.12 ± 66.51 | NR |
| Tenofsky et al. [57] | Preferred ≥2 mm; no cancer at skin/muscle edge | NR | NR | 0/58 (0)† | 0/84 (0)† | 3/58 (5.2)† | 11/84 (13.1)† | NR | NR | NR | NR | 3/58 (5.2) | 11/84 (13.1) | NR | NR | 3/58 (5.2) | 11/84 (13.1) | 0/58 (0) | 1/84 (1.2) | NR | NR | NR; component events reported† | NR; component events reported† | Component complications reported separately, including fat necrosis and non-healing wounds; no patient-level overall complication denominator. Severity: formal grading NR. | 56.0 ± 20.4 | 51.1 ± 23.2 | Cosmetic complaint: 8/58 (13.8) vs 6/84 (7.1) |
| Tokui et al. [58] | Invasive or noninvasive component at inked margin | 6/145 (4.1) | 112/1040 (10.8) | 4/145 (2.8) | 76/1040 (7.3) | NR | NR | 139/145 (95.9) | 928/1040 (89.2) | NR | NR | 2/145 (1.4) | 36/1040 (3.5) | 0/145 (0) | 16/1040 (1.5) | 2/145 (1.4) | 52/1040 (5.0)† | NR; 5-y LRFS 98% | NR; 5-y LRFS 98% | NR | NR | NR | NR | NR | NR | NR | NR |
| Vieira et al. [59] | Free margins; margin distance reported | 0/26 (0)† | 0/52 (0)† | 0/26 (0)† | 0/52 (0)† | NR | NR | 26/26 (100)† | 52/52 (100)† | 16.44 ± 8.64 mm | 11.85 ± 8.36 mm | 0/26 (0)† | 0/52 (0)† | 0/26 (0)† | 0/52 (0)† | 0/26 (0)† | 0/52 (0)† | 2/26 (7.7) | 6/52 (11.5) | 3/26 (11.5) | 7/52 (13.5) | NR | NR | NR | NR | NR | NR |
| Vohra et al. [60] | No tumor cells on inked surface | 6/277 (2.2) | 32/204 (15.7) | NR | NR | NR | NR | 271/277 (97.8) | 172/204 (84.3) | NR | NR | 0/277 (0) | 17/204 (8.3) | 4/277 (1.4) | NR | 4/277 (1.4) | 17/204 (8.3)† | NR | NR | NR | NR | 6/277 (2.2) | 0/204 (0) | Types: seroma; fat necrosis; wound infection. Severity: reported complications did not require operative intervention; formal grading NR. | NR | NR | NR |
| Wijesinghe et al. [61] | Close <1 mm/positive margin requiring re-excision | NR | NR | NR | NR | NR | NR | NR | NR | 16 (4–25) mm | 6 (<1–12) mm | 1/73 (1.4) | 9/54 (16.7) | NR | NR | 1/73 (1.4) | 9/54 (16.7) | NR | NR | NR | NR | 3/73 (4.1) | 1/54 (1.9) | Type: wound dehiscence. Severity: Clavien-Dindo classification used; reported complication was Clavien-Dindo grade I. | NR | NR | PROM better with O-BCS: 10/9.8/9.2/9.4 vs 8/8.5/6/5.6 |
| Zhou et al. [62] | Intraoperative frozen-section positive margin | 2/43 (4.65) | 11/63 (17.46) | NR | NR | NR | NR | 41/43 (95.35) | 52/63 (82.54) | NR | NR | 1/43 (2.33)† | 0/63 (0)† | NR | 1/63 (1.59) | 1/43 (2.33) | 1/63 (1.59) | 0/43 (0)† | 0/63 (0)† | NR | NR | 0/43 (0) | 0/63 (0) | No short-term postoperative complications observed in either group. Severity classification: NA. | NR | NR | High satisfaction 95.35% vs 96.83%; excellent 81.40% vs 87.30% |
| Note: Data are shown as n/N (%) unless otherwise indicated. Initial PM was prioritized for margin-status synthesis unless only final PM was reported.  Complication laterality was recorded when reported; most studies did not distinguish index-breast from contralateral symmetrization-related events.  † Non-standard, derived, surrogate, intraoperative, combined-group, or internally inconsistent outcome/denominator; handle in sensitivity analysis when used for pooling.  ‡ Definition differs from the pure category or represents a composite category as reported in the original study.  § Focally positive margin category under the original guideline. Complication types and severity classifications were recorded as reported in the original studies. Clavien-Dindo grades were retained only when explicitly reported; no retrospective severity grading was assigned when information required for reliable classification was unavailable.  Abbreviations: BC, breast cancer; BCS, breast-conserving surgery; C-BCS, conventional breast-conserving surgery; BREAST-Q, Breast-Q questionnaire; FS, frozen section; CM, completion/conversion mastectomy; DCIS, ductal carcinoma in situ; IBTRFS, ipsilateral breast tumor recurrence-free survival; LR, local recurrence; LRR, locoregional recurrence; MRR, margin-related reoperation; NA, not applicable; NR, not reported; OBCS, oncoplastic breast-conserving surgery; OR, operating room; PM, positive margin; PROM, patient-reported outcome measure; RE, re-excision; RT, radiotherapy; SD, standard deviation; SEM, standard error of the mean. | | | | | | | | | | | | | | | | | | | | | | | | | | | |
