# Supplementary material for: Margin status, re-excision, and completion mastectomy after oncoplastic versus conventional breast-conserving surgery for breast cancer: a systematic review and meta-analysis
Source: Front Oncol. 2026 Sep 15;16:1934279. doi: 10.3389/fonc.2026.1934279 (PMC13619401; doi:10.3389/fonc.2026.1934279)
Supplement: Supplementary file 4 [file Table3.docx]

| Supplementary Table S3. Sensitivity analyses for meta-analytic outcomes | | | | | | | | | | | | | | | | |
| --- | --- | --- | --- | --- | --- | --- | --- | --- | --- | --- | --- | --- | --- | --- | --- | --- |
| Effect measure: risk ratio (RR), Mantel–Haenszel random-effects model. The direction of effect favors OBCS when RR < 1 for adverse outcomes and RR > 1 for negative/clear margin. | | | | | | | | | | | | | | | | |
| Outcome | Outcome category | Main analysis studies | Main OBCS events/total | Main C-BCS events/total | Main RR (95% CI) | Main P value | Main I² | Sensitivity analysis | Studies changed/excluded/added | Sensitivity OBCS events/total | Sensitivity C-BCS events/total | Sensitivity RR (95% CI) | Sensitivity P value | Sensitivity I² | Figure location | Result-level interpretation |
| Initial positive surgical margin | Primary margin-related outcome | 19 comparisons | 159/2,054 | 748/6,518 | 0.57 (0.40–0.80) | 0.001 | 61% | Overlap-cohort replacement | Vohra et al. [60] replaced by Abidi et al. [18] | 158/2,026 | 747/6,487 | 0.56 (0.40–0.80) | 0.001 | 62% | Supplementary Fig. S1 | No material change in effect direction, magnitude, or statistical significance. |
| Re-excision | Primary margin-related outcome | 29 comparisons | 1,324/19,085 | 2,536/26,615 | 0.59 (0.46–0.77) | <0.0001 | 60% | Overlap-cohort replacement | Vohra et al. [60] replaced by Abidi et al. [18] | 1,326/19,057 | 2,536/26,584 | 0.58 (0.45–0.75) | <0.0001 | 62% | Supplementary Fig. S2 | No material change in effect direction, magnitude, or statistical significance. |
| Completion/conversion mastectomy | Primary margin-related outcome | 20 comparisons | 917/17,569 | 1,151/22,070 | 0.62 (0.43–0.90) | 0.01 | 63% | Definition/data-coding caveat inclusion | Crown et al. [33] and Nisiri et al. [51] added | 931/18,052 | 1,168/22,514 | 0.65 (0.46–0.91) | 0.01 | 60% | Supplementary Fig. S3 | Direction and statistical significance were preserved. |
| Completion/conversion mastectomy | Primary margin-related outcome | 20 comparisons | 917/17,569 | 1,151/22,070 | 0.62 (0.43–0.90) | 0.01 | 63% | Overlap-cohort replacement | Falade et al. [38] replaced by Mukhtar et al. [48] | 914/17,489 | 1,147/22,021 | 0.66 (0.47–0.94) | 0.02 | 56% | Supplementary Fig. S4 | Direction and statistical significance were preserved; heterogeneity was slightly reduced. |
| Any margin-related reoperation | Primary margin-related outcome | 27 comparisons | 2,110/18,749 | 3,340/25,533 | 0.63 (0.51–0.78) | <0.0001 | 63% | Registry/database study exclusion | André et al. [21] and Kilmer et al. [44] excluded | 243/3,157 | 838/6,679 | 0.55 (0.41–0.73) | <0.0001 | 63% | Supplementary Fig. S5 | Direction and statistical significance were preserved after excluding large registry/database studies. |
| Close margin | Secondary margin-status outcome | 6 comparisons | 25/301 | 103/698 | 0.67 (0.39–1.16) | 0.16 | 33% | Broader/non-standard definition inclusion | Bargon et al. [13], Down et al. [37], Nisiri et al. [51], Oh et al. [52], and Tenofsky et al. [57] added | 95/1,211 | 372/3,065 | 0.74 (0.55–1.01) | 0.06 | 30% | Supplementary Fig. S6 | Effect direction remained similar; the result remained statistically non-significant. |
| Negative/clear margin | Secondary margin-status outcome | 19 comparisons | 1,924/2,103 | 5,757/6,568 | 1.07 (1.04–1.11) | <0.0001 | 71% | Overlap-cohort replacement | Vohra et al. [60] replaced by Abidi et al. [18] | 1,897/2,075 | 5,727/6,537 | 1.07 (1.04–1.11) | <0.0001 | 72% | Supplementary Fig. S7 | No material change in effect direction, magnitude, or statistical significance. |
| Negative/clear margin | Secondary margin-status outcome | 19 comparisons | 1,924/2,103 | 5,757/6,568 | 1.07 (1.04–1.11) | <0.0001 | 71% | Broader/non-standard definition inclusion | Di Micco et al. [36], Down et al. [37], Kaur et al. [42], Oh et al. [52], Rose et al. [53], and Vieira et al. [59] added; Abidi/Falade/Mukhtar not added to avoid overlap mixing | 2,593/2,827 | 7,474/8,593 | 1.07 (1.04–1.11) | <0.0001 | 73% | Supplementary Fig. S8 | Direction and statistical significance were preserved; heterogeneity remained substantial. |
| Local recurrence / IBTR | Oncologic safety outcome | 18 comparisons | 65/2,839 | 244/7,996 | 0.84 (0.64–1.11) | 0.22 | 0% | No additional sensitivity analysis performed | No predefined overlap or definition-caveat sensitivity set was used for this outcome; statistical heterogeneity was not observed | NA | NA | NA | NA | NA | Not applicable | Main analysis showed no statistically significant difference and no observed statistical heterogeneity. |
| Locoregional recurrence | Oncologic safety outcome | 10 comparisons | 36/1,571 | 113/3,448 | 0.75 (0.51–1.11) | 0.15 | 0% | No additional sensitivity analysis performed | No additional studies with predefined sensitivity or overlap flags were available; statistical heterogeneity was not observed | NA | NA | NA | NA | NA | Not applicable | Main analysis showed no statistically significant difference and no observed statistical heterogeneity. |
| Overall complications | Surgical safety outcome | 16 comparisons | 4,769/18,412 | 10,580/69,385 | 1.40 (1.02–1.92) | 0.04 | 95% | Registry/database study exclusion | Kilmer et al. [44] and Shah et al. [56] excluded | 175/1,726 | 322/2,638 | 1.23 (1.00–1.50) | 0.05 | 10% | Supplementary Fig. S9 | Effect estimate was attenuated and heterogeneity was markedly reduced after excluding registry/database studies. |
| Notes: 1. Main analyses were based on the predefined main-analysis dataset for each outcome. Sensitivity analyses were selected according to overlap-cohort concerns, broader/non-standard outcome definitions, or registry/database-derived outcome ascertainment. 2. For adverse outcomes, including positive margin, re-excision, completion/conversion mastectomy, any margin-related reoperation, close margin, recurrence, and complications, RR < 1 favors OBCS. For negative/clear margin, RR > 1 favors OBCS because the event is favorable. 3. NA indicates that no additional sensitivity analysis was performed or that no sensitivity estimate was applicable. Abbreviations: BCS, breast-conserving surgery; C-BCS/CBCS, conventional breast-conserving surgery; CI, confidence interval; IBTR, ipsilateral breast tumor recurrence; I², inconsistency statistic; MRR, margin-related reoperation; OBCS, oncoplastic breast-conserving surgery; RR, risk ratio. | | | | | | | | | | | | | | | | |
